# Single-Residue Mutation Switch Reconfigures the Hierarchical Structure and Assembly of Amphiphilic Protein Block Copolymers for Hydration Layer-Dominated Water-Responsive Actuation

**DOI:** 10.1021/acs.biochem.6c00038

**Published:** 2026-06-22

**Authors:** Jonathan W. Sun, Chengyu Sun, Seungri Kim, Andrew L. Wang, Isabella Huang, Nada Haq-Siddiqi, Zara Hedaya, Raymond S. Tu, Xi Chen, Jin Kim Montclare

**Affiliations:** † Department of Chemical and Biomolecular Engineering, 34242 New York University (NYU) Tandon School of Engineering, Brooklyn, New York 11201, United States; ‡ Department of Chemistry, New York University, New York, New York 10003, United States; § Department of Chemical Engineering, 449003 City College of New York, New York, New York 10031, United States; ∥ Department of Biomedical Engineering, State University of New York (SUNY) Downstate Health Sciences University, Brooklyn, New York 11203, United States; ⊥ Advanced Science Research Center (ASRC) at the Graduate Center, City University of New York, New York, New York 10031, United States; # PhD Programs in Chemistry and Physics at the Graduate Center, City University of New York, New York, New York 10016, United States; ∇ Department of Biomedical Engineering, NYU Tandon School of Engineering, Brooklyn, New York 11201, United States; ○ Department of Radiology, NYU Langone School of Medicine, New York, New York 10016, United States; ◆ Department of Biomaterials, NYU College of Dentistry, New York, New York 10010, United States

## Abstract

Protein folding dynamics emerge from subtle changes in intramolecular interactions, where hydration shapes free energy landscapes and influences conformational ensembles and supramolecular assembly. While these shape-shifting principles have been primarily explored in well-behaved systems with sharp transitions, their applicability to multidomain protein assemblies, particularly those containing partially folded or disordered regions, remains unclear. Here, we investigate how a single leucine-to-alanine mutation modulates the hierarchical interplay between folding, micelle formation, and macroscopic biomaterial behavior using two diblock protein block copolymers (BCPs): CE and C_L44A_E. In solution, both BCPs exhibit spectral features consistent with their C/C_L44A_ monoblocks, with CE showing α-helical features and C_L44A_E more disordered ones. Upon dehydration, however, both systems display increased structural order, with CE favoring β-sheets and C_L44A_E adopting α-helical content. At the mesoscale, both BCPs assemble into amphiphilic micelles, but CE forms smaller, densely packed micelles, whereas C_L44A_E generates larger micelles that template film networks of varying pore size depending on their packing density. These differences lead to distinct solvation behavior, with C_L44A_E exhibiting greater uptake of predominantly mobile water, approximately twice that of CE, culminating in an actuation energy density of 2,043 kJ/m^3^, the highest reported for protein BCPs to date. Despite this increased uptake, C_L44A_E shows reduced mechanical relaxation and a rougher DSC/TGA thermogram, indicative of a broader conformational ensemble compared to CE. Comparisons to monoblocks (C, C_L44A_, E) and previously reported triblocks (CEC, C_L44A_EC_L44A_) further highlight the importance of asymmetric diblock architectures in promoting hydration-sensitive assemblies, laying the rational design groundwork for future high-performance, water-responsive protein biomaterials.

## Graphical abstract

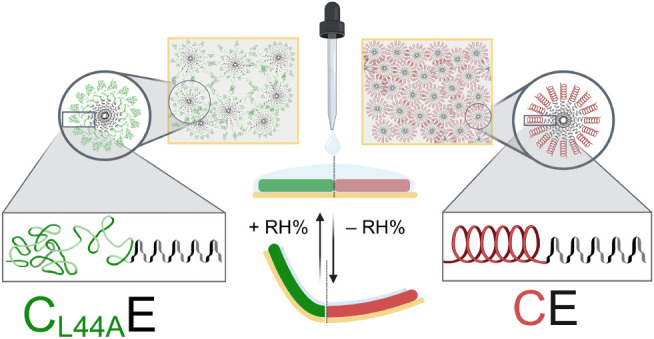

## 1 Introduction

From coordinated contraction of muscles^1^ to aiding transport along microtubules,^2^ dynamic motion in biological systems is facilitated by specialized proteins operating as pseudoisothermal machines. These systems convert chemical energy directly into mechanical work through reversible folding and unfolding cycles, analogous to the expansion and contraction phases of thermodynamic engines.^3,4^ Recent engineering efforts have sought to harness these principles in the development of water-responsive (WR) biomaterials, where changes in environmental hydration via relative humidity (RH) drive large-scale shape changes, including swelling, contraction, or bending.^5–8^ By utilizing the chemical potential of evaporative water under ambient conditions, these biomaterials can bypass the energetic penalties or auxiliary power requirements of conventional energy-harvesting solutions that can offset net energetic gains.^4,9,10^ To achieve such efficiency, natural systems typically employ multidomain, amphiphilic architectures, composed of distinct hydrophilic and hydrophobic regions, that can amplify nanoscale changes in hydration into macroscopic motion. For instance, the seed capsules of ice plants (i.e., *Aizoaceae*) utilize bilayer keel structures, where an active hygroscopic inner layer expands against a stiffer, less-responsive outer layer to open the pod in high RH environments.^11^ Similarly, filaree (i.e., *Erodium cicutarium*) seeds harness dehydration-driven contractions within their helical cellulose fiber network to generate a coordinated twisting motion for soil embedding.^12^ Spider dragline silk has emerged as a benchmark WR biomaterial, where its hydrophobic β-sheet domains embedded within a disordered, glycine-rich hydrophilic network enable cyclic stresses approaching 80 MPa.^13^ Infiltration of water disrupts its internal hydrogen bonded (H-bonded) network, driving structural reorganization and yielding impressive WR energy densities near 500 kJ/m^3^, orders of magnitude higher than mammalian muscle (∼8 kJ/m^3^).^6,13^ These advances position WR biomaterials as promising active components in soft robotics,^14,15^ energy-harvesting devices,^10^ smart textiles,^16–18^ and biosensors.^19,20^

Despite growing interest in these systems, emulating this efficiency in synthetic materials requires comprehensive understanding of a protein’s free energy landscape. While protein designers typically emphasize encoding specific folded structures through the primary amino acid sequence, the hydration state of the system also plays an impactful role. Computational molecular dynamics simulations with explicit water suggest that as a protein undergoes conformational transitions, some water remains tightly associated with the protein surface in a “hydration shell,” which serves to modulate the balance of thermodynamic forces governing structural stabilization (e.g., solvent entropy, H-bonding, hydrophobic collapse).^21–24^ As water is progressively removed through desolvation or reintroduced through resolvation, the free energy landscape is fundamentally reshaped, lowering energetic activation barriers and allowing a broader ensemble of partially hydrated, partially folded, metastable conformations to emerge.^23–25^ Thus, the thermodynamic stability of protein states and the kinetics governing structural transitions are inextricably linked to the competitive balance between protein–protein packing and protein–solvent interactions.

Beyond theoretical models, however, empirical evidence for structural hydration shells has also emerged from experimental studies of dipeptide (i.e., GS, GT, GH, AH, LH, YH) and tripeptide (i.e., HYF, DYF, YFD) supramolecular crystals. In these systems, discrete water-binding nanoscale channels induce dynamic lattice contractions and expansions during partial or complete desolvation and resolvation, driving rapid and reversible WR actuation.^26–28^ However, unlike these perfectly ordered crystalline systems, the hydration-driven mechanical response in softer, longer protein-based architectures remains significantly less understood. This gap is particularly pronounced for constructs containing intrinsically disordered or partially structured domains, where traditional protein folding models fail to capture the broader conformational ensembles accessible under varying hydration conditions.^29^

One approach to bridging this gap is to combine ordered and disordered protein domains within a single construct, enabling controlled interplay between distinct, structure-based assembly modes. To this end, our group has developed a series of protein block copolymers (BCPs) that fuse two self-assembling domains (SADs): α-helical coiled-coil (C) domains derived from cartilage oligomeric matrix protein (COMP) and elastin-like polypeptides (E) composed of (VPGXG)_*n*_ repeats, where X is valine or phenylalanine (Table S1).^30–35^ This segmented, modular architecture allows each folded domain to be treated as a block that retains most of its intrinsic secondary structure and physiochemical character.

In prior work, we examined two triblock BCP constructs, CEC and C_L44A_EC_L44A_, to begin exploring how hydration hierarchically influences structure and assembly. Under ambient conditions, the E domain undergoes spontaneous coacervation, driving the formation of micellar assemblies with elastin-rich cores and coiled-coil rich coronas.^32,34^ When cast into films, CEC achieved a moderate actuation energy density (119.4 ± 34.3 kJ/m^3^), however, disruption of α-helical coiled-coil interactions in the terminal C domains via two leucine-to-alanine mutations at the 44th position (C_L44A_) to yield C_L44A_EC_L44A_ resulted in a 5.4-fold enhancement in WR energy density (647.9 ± 86.8 kJ/m^3^).^33^

Through systematic structural, supramolecular, and bulk materials characterization, we demonstrate how sequence-level mutations give rise to distinct structural responses upon desolvation, which translate hierarchically into differences in supramolecular micelle assembly and hydration behavior. CE forms compact, densely packed micelles (*D*_H_: ∼52.0 nm) that dry and preorganize smooth films, whereas C_L44A_E assembles into larger swollen micelles (*D*_H_: ∼190.6 nm) that give rise to highly porous networks. These differences in assembly additionally govern both the magnitude and association strength of the hydration layer. C_L44A_E absorbs 128% more water mass compared to CE, but this water is predominantly mobile and weakly associated. By contrast, CE incorporates less water overall, but supports more strongly associated hydration within a more tightly confined and cooperative interfacial environment. These distinctions in hydration layer interactions are further reflected in the hydration-dependent mechanical response of the bilayer actuator films. Despite greater overall water uptake, C_L44A_E retains higher stiffness under hydrated conditions, whereas CE undergoes more pronounced softening. Enhanced water sorption in tandem with BCP shape change underlies the exceptionally high macroscopic WR actuation energy density of C_L44A_E at 2,043 kJ/m^3^, exceeding CE’s WR actuation energy density (481.4 kJ/m^3^) by more than 4-fold and surpassing previously reported triblock protein BCP systems.^27^ Together, these findings demonstrate that a single-point mutation can reprogram protein behavior across hierarchical length scales, coupling local secondary structure, supramolecular assembly, hydration layer interactions, and ultimately macroscopic actuation, establishing sequence-level control as a powerful means of simultaneously tuning protein–protein and protein–solvent interactions in WR protein biomaterials.

## 2 Materials and Methods

### 2.1 General Materials and Reagents

Phenylalanine auxotrophic derivatives of *Escherichia coli* (*E. coli*) BL21­(DE3) strain bearing the repressor plasmid pLysS-IQ (AF-IQ) were a gift from David Tirrell (California Institute of Technology).^36^ Bactotryptone, sodium chloride, yeast extract, tryptic soy agar, ampicillin sodium salt (AMP), chloramphenicol (CAM), HisPur Nickel-nitrotriacetic acid (Ni-NTA) agarose resin, sodium hydroxide (NaOH), isopropyl β-d-1-thiogalactopyranoside (IPTG), Tris­(hydroxymethyl)­aminomethanehydro chloride (Tris-HCl), imidazole, urea, Coomassie Brilliant Blue R-250 dye, sodium dodecyl sulfate (SDS), sodium phosphate dibasic salt (Na_2_HPO_4_), sodium phosphate monobasic salt (NaH_2_PO_4_), potassium phosphate monobasic (KH_2_PO_4_), ammonium chloride (NH_4_Cl), methanol, acetic acid, acetonitrile (ACN), bicinchoninic acid (BCA) assay kit, bovine serum albumin (BSA) standard ampules, SnakeSkin dialysis tubing with 3.5 and 10 kDa molecular weight cut off (MWCO) were obtained from Thermo Fisher Scientific. Yeast extract, d-(+)-glucose, magnesium sulfate (MgSO_4_), calcium chloride (CaCl_2_), manganese chloride (MnCl_2_), cobalt chloride (CoCl_2_), zinc sulfate (ZnSO_4_), copper chloride (CuCl_2_), nickel chloride (NiCl_2_), sodium selenite (Na_2_SeO_3_), boric acid (H_3_BO_3_), sodium molybdate (Na_2_MoO_4_), iron chloride (FeCl_3_), thiamine hydrochloride (VitB), α-cyano-4-hydroxycinnamic acid (CHCA), sinapinic acid (SA), trifluoroacetic acid (TFA) were purchased from Sigma-Aldrich (St. Louis, MO). α-d-Lactose monohydrate was purchased from Acros Organics. Bacteriological-grade tryptone and hydrochloric acid (HCl) were purchased from VWR. 0.22 μm syringe filters, Macrosep and Microsep advanced centrifugal devices with 3.5 or 10 kDa MWCO were purchased from Pall Corporation. *Kpn*I restriction enzyme, *Hin*dIII restriction enzyme, and T4 DNA ligase were purchased from New England Biolabs. ZymoClean Gel DNA Extraction Kit was purchased from Zymogen. Acrylamide/bis solution (30%) 29:1 and Precision Plus Protein unstained protein standards were purchased from Bio-Rad. Polyimide (PI) solid support with a thickness of 14 μm was obtained from CAPLINQ and cut to 3 × 6 mm strips before use.

### 2.2 Molecular-Level Characterization

#### 2.2.1 Matrix-Assisted Laser Desorption Ionization-Time of Flight Mass Spectrometry (MALDI-ToF MS)

To provide a higher resolution of the synthesized protein BCPs, MALDI-ToF MS was performed on intact protein samples. Postdialysis samples underwent zip-tip desalting using Pierce C18 tips with a 10 μL bed volume (Millipore, Billerica, MA). Samples were eluted in 80% ACN diluted with a 0.1% solution of TFA in water and mixed with an equal volume of supersaturated CHCA (for small monoblock proteins ≤ 10 kDa) or SA (for protein BCPs > 10 kDa) matrix solution. Using the dried droplet method, equal volumes of the sample and the supersaturated CHCA or SA matrix solution (25 mg in 1 mL of 50:50 ACN:water with 0.1% TFA) were mixed and deposited onto an MTP 384 target plate made of ground steel (Bruker Daltonics, Billerica, MA) and desiccated under vacuum for at least an hour.^37^ Measurements were performed on the autoflex maX (Bruker Daltonics, Billerica, MA). Calibration of the system was completed before each measurement using the ProteoMass Peptide and Protein MALDI MS calibration kit (Sigma-Aldrich, St. Louis, MO) at working solutions of 10 pmol/μL. Average mass spectra of at least 500 laser shots were acquired in linear laser mode with a power of at least 80% equipped with a high-energy pulse of >85 μJ at 1 kHz. Spectra were baselined, overlaid, and processed in mMass software.^38^ Peaks were identified using the mMass peak detection algorithm, applying a signal-to-noise (S/N) threshold of 4.0. Quantitative peak parameters, including their integration values, S/N ratio, and full width at half-maximum (fwhm) are detailed in Table S2 alongside their assignments and agreement with theoretical molecular masses.

#### 2.2.2 Circular Dichroism (CD) Spectroscopy

The secondary structure of each sample in the solution state was ascertained using a J-815 Circular Dichroism spectrophotometer equipped with a PTC-423S single-position Peltier temperature control system (Jasco, Easton, MD). A quartz cuvette (Hellma Analytics, Plainview, NY) with a 1 cm path length was loaded with each protein solution diluted to 10 μM in pure deionized water. Wavelength scans were collected at 20 °C from 190 to 250 nm at a rate of 1 nm/s intervals. Following spectral acquisition, samples were extracted from the cuvette and subjected to BCA assay to assess the actual concentration of the sample for comparative quantitative analysis. Spectra were background subtracted, smoothed using a Savitzky–Golay filter,^39^ and converted to mean residual ellipticity before secondary structure composition was estimated using the BeStSel algorithm (Version 1.3.230210).^40^

#### 2.2.3 Fourier-Transform Infrared (FTIR) Spectroscopy for Protein Secondary Structure

FTIR spectroscopy was performed using a Nicolet iS50 FTIR Spectrometer equipped with a DTGS-KBr detector (Thermo Scientific, Waltham, MA). After blanking under ambient atmosphere, 10–20 mg of lyophilized protein was deposited onto the CdTe stage and allowed to equilibrate for 20 min under ambient lab conditions ambient laboratory conditions (*T* = 25 °C, RH ∼ 20%). Spectra were acquired, using 64 scans at a spectral resolution of 4 cm^–1^. Analysis focused on the amide I region (ν̃ = 1600–1700 cm^–1^) for protein secondary structural determination in the lyophilized state. Subpeak deconvolution was performed by fitting nonlinear Gaussian models in PeakFit (Systat Software, Inc.) with secondary structure assignments made in accordance with the Jackson model.^41,42^

#### 2.2.4 FTIR Spectroscopy for Hydration Layer Characterization

Transmission FTIR spectroscopy was performed using a Nicolet Summit X spectrometer (Thermo Scientific, Waltham, MA), equipped with a DTGS-KBr detector and a customized RH-controlled chamber with CaF_2_ windows. Lyophilized protein was reconstituted in DI water to prepare a 10 mg/mL solution. A 200 μL aliquot was cast onto CaF_2_ windows and allowed to air-dry overnight under ambient laboratory conditions (*T* = 25 °C, RH ∼ 20%). Spectra were collected using 64 scans at a spectral resolution of 4 cm^–1^, while RH was systematically varied during measurements. To minimize environmental variability, all measurements were conducted on the same day under identical conditions. Analysis focused on the O–H stretching region (ν̃ ≈ 2,700–3,700 cm^–1^) to characterize H-bonding. Subpeak deconvolution was performed using nonlinear Gaussian fitting with fwhm constrained to 20–30 cm^–1^ in OriginPro (Version 2023b, Northampton, MA), and water populations were assigned based on the Podbevšek model.^42^

### 2.3 Supramolecular Assembly Characterization

#### 2.3.1 Dynamic Light Scattering (DLS)

The hydrodynamic size of assembled protein BCPs was determined from DLS measurements performed on a Malvern Zetasizer Nano ZS90 (Malvern Instruments, Malvern, UK). Both sets of measurements were performed at 25 °C using a standard protein material reflective index of 1.450. Water was used as the dispersed phase for DLS analysis with a refractive index of 1.330 and a viscosity of 0.8872 cP. DLS measurements were performed in disposable clear square cuvettes with standard Mark–Houwink parameters (*A*: 0.428 and *K*: 7.67 × 10^–5^ cm^2^/s) with a 4 mW helium–neon laser (λ = 633 nm) and a 90° backscatter detector. Three independent protein samples with at least 10 measurements per sample, 10 runs per measurement. After 120 s of equilibration time, the particle count rate, correlation function *G­(τ)*, and intensity-weighted particle size distribution were acquired using the Zetasizer software (Version 6.32) following standard photon correlation spectroscopy theory.^43^ Specifically, the hydrodynamic diameter (*D*_H_) distribution of the assembled particles was calculated as a distribution of relaxation time (τ) using the Stokes–Einstein relation:

1 $$D_{H}\left(\tau \right)=\frac{k_{B}T\;q^{2}}{3\pi \eta \Gamma }$$

where *k*_B_ is the Boltzmann constant, *T* is the temperature, *η* is the viscosity of the solvent, *Γ* is the decay rate of the second-order correlation function *G*″(*τ*), and *q* is the magnitude of the scattering vector.

#### 2.3.2 Static Light Scattering (SLS)

SLS measurements were performed on varied concentrations of protein BCP at 25 °C in a clear, square Q quartz cuvette with a circular aperture (Malvern Panalytical, Westborough, MA) with a θ = 173° backscatter detector to estimate protein–solvent interactions and the molecular weight (*M*_w,assembly_) of the assemblies. Assuming a random coil shape correction (*R*_g_/*R*_h_ = 0.816), a Debye plot was constructed and linearized to fit the simplified Debye–Zimm equation using Minitab (Version 22.2.2):

2 $$\frac{Kc}{R_{\theta }}=\frac{1}{M_{w,\mathrm{assembly}}}+2A_{2}c$$

where *c* is the concentration determined from BCA (in mg/mL), *R*_θ_ is the Rayleigh ratio or the normalized scattered intensity (toluene with a reflective index of 1.386 and a viscosity of 1.10 cP was applied as the standard solvent), *A*_2_ is the second Virial coefficient, and *K* is the optical constant.

The aggregation number (*N*_agg_) was estimated as the ratio of *M*_w,assembly_ to the molecular weight of each diblock (*M*_w,diblock_):

3 $$N_{\mathrm{agg}}=\frac{M_{w,\mathrm{assembly}}}{M_{w,\mathrm{diblock}}}$$

#### 2.3.3 Transmission Electron Microscopy (TEM)

TEM was used to characterize the morphology of the protein BCP assemblies. To mimic WR actuator casting conditions, 10 μL of protein samples at 10 mg/mL in deionized water were spotted onto a 400-mesh Formvar/carbon-coated copper grid (Electron Microscopy Sciences, Hatfield, PA) and dried overnight at ambient conditions. A 1% (w/v) uranyl acetate solution (Electron Microscopy Sciences, Hatfield, PA) was used as the negative stain. Specifically, 5 μL of the stain was applied and immediately blotted twice, followed by a final application of 5 μL of the stain, which was allowed to incubate for 5 min to ensure optimal contrast. Excess stain was gently removed using Whatman filter paper (Whatman (Cytiva), Marlborough, MA) and promptly air-dried. Samples were imaged using an FEI Talos L120C TEM equipped with a Gatan 4k × 4k OneView digital camera. Micrographs were processed and analyzed using ImageJ software (Version 1.8.0, NIH, Bethesda, MD) to ensure precise morphological characterization. The dry Feret diameters (*D*_D_), defined as the maximum distance between two parallel tangents of the assembly projection, were recorded for *n* = 300 assemblies (Figure S3). Assuming spherical geometry and isotropic shrinkage during drying, the volumetric swelling ratio (*Q*_v_) and protein volume fraction (ϕ) within the micelle assemblies were estimated using

4 $$Q_{v}=\frac{1}{\phi }={\left(\frac{D_{H}}{D_{D}}\right)}^{3}$$

### 2.4 Solid-State Film Fabrication and Characterization

#### 2.4.1 Preparation of BCP/PI Bilayer Actuators

BCP/PI bilayer actuators were fabricated by depositing 7 μL of protein BCP suspension (10 mg/mL in deionized water) onto plasma-treated PI substrates. Bilayer films were then dried overnight at room temperature. Actuation curvature was assessed at RH 10% and 90% in a RH-controlled chamber. Curvature (Γ) was extracted using ImageJ by fitting the actuator profile to a circular arc. The actuation energy density (*U*) was calculated following:

5 $$U=\frac{E_{1}I_{1}+E_{2}I_{2}}{Lbt\Gamma^{2}}$$

where Γ is the radius of curvature, *E*_1_ and *E*_2_ are the Young’s moduli of each BCP (7.3 GPa for C_L44A_E and 7.6 GPa for CE) and PI substrate (2.5 GPa), respectively, *I*_1_ and *I*_2_ are their respective area moments of inertia, and *L*, *b*, and *t* denote the length, width, and thickness of the polymer layer. Full expressions for geometric terms and sensitivity analysis are provided in the Supporting Methods and Figure S12.

#### 2.4.2 Environmental Scanning Electron Microscopy (ESEM)

Surface morphologies of the bilayer films under controlled RH conditions were examined using a Quattro S environmental scanning electron microscope (Thermo Scientific, Waltham, MA). Samples were mounted on a temperature-controlled stage using conductive adhesive and imaged without conductive coating to preserve their native hydrated morphology. The chamber RH was regulated by controlling the water vapor pressure at constant temperature (i.e., 4 °C), and samples were allowed to equilibrate for at least 5 min at each RH prior to imaging. Images were acquired in environmental mode using a gaseous secondary electron detector at an accelerating voltage of 10–15 kV to minimize beam-induced dehydration and damage.

#### 2.4.3 Young’s Modulus Measurements

The Young’s modulus of BCP was characterized by a customized Multimode 8 Atomic Force Microscope (AFM) (Bruker, Billerica, MA) under pseudoisothermal conditions held at 25 °C. A 10 mg/mL solution of BCP was first deposited onto a silicon wafer. After drying overnight at room temperature, a layer with a thickness of 3 μm was formed. To control the local RH, the flow rates of humid and dry air were adjusted until the RH stabilized at the desired level for at least 5 min, monitored by a commercial HIH-4021 RH sensor (Honeywell, Charlotte, NC). Once the RH reached a stable level, the topography of the samples was measured using a SCANASYST-AIR AFM probe (Bruker, Billerica, MA) and analyzed using the Nanoscope Analysis software (Version 1.90, Bruker, Billerica, MA). AFM nanoindentation measurements were used to quantify the Young’s modulus of each BCP under controlled RH. NCHV probes (Bruker, Billerica, MA) with a spring constant of 33.8 N/m were employed. Young’s moduli were extracted from force–distance curves (Figure S11) using the following Hertzian contact model, which assumes purely elastic, nonadhesive contact between a rigid indenter and a homogeneous, isotropic sample:^7^

6 $$F^{2/3}={\left(\frac{4}{3}\frac{E}{{\left(1-v\right)}^{2}}\sqrt{R}\right)}^{2/3}d$$

where *F* is the indentation force, *E* is the Young’s modulus, *v* is the Poisson’s ratio (0.32) for semihydrated protein assemblies,^44^ *R* is the probe radius (8 nm), and *d* is the indentation depth.

To satisfy these model assumptions, we limited indentations to small depths relative to the tip radius. Moreover, the disparity in stiffness between the NCHV tip (120 GPa) and the BCP films justifies the rigid indenter approximation.^45^

#### 2.4.4 Water Vapor Sorption Measurements

Water sorption isotherms were measured using dynamic vapor sorption (DVS) conducted using a DVS Intrinsic system (Surface Measurement Systems, Allentown, PA) at 25 °C. Films cast with 2 mg of dried protein BCP were tested under RH cycling from ∼8% to 86%. Each RH step was held for a minimum of 60 min or until the mass change rate stabilized below 0.0005 mg/min for 10 consecutive min. Water sorption isotherms were obtained over three full RH cycles. The mass percent vapor sorption (*%VS*) at each RH was calculated as

7 $$\%\mathrm{VS}=\frac{\left(m-m_{8\%RH}\right)}{m_{8\%RH}}$$

where *m* is the sample mass at a given RH and *m*_8%RH_ is the reference mass of each protein BCP at 8% RH.

Using OriginPro (Version 2023b, Northampton, MA), the resulting water sorption isotherms were fitted to the Guggenheim–Anderson–de Boer (GAB) model:

8 $$\frac{m}{m_{mG}}=\frac{c_{G}ka_{w}}{\left(1-ka_{w}\right)\left(1-ka_{w}+c_{G}ka_{w}\right)}$$

where *a*_w_, the water activity, is defined as the ratio of water vapor pressure to the saturated water pressure in solution (*p*/*p*_0_), *m*_mG_ is the moisture content of the monolayer by mass, *c*_G_ is GAB constant related to the difference in sorption enthalpy, and the dimensionless constant *k* accounts for the thermodynamic properties of multilayer water relative to bulk liquid water.^46,47^

For CE, both sorption and desorption isotherms were fitted over an RH range of 8–86%. For C_L44A_E, sorption and desorption isotherms were fit separately using data below and above 68% RH, respectively, to distinguish different modes of water sorption behavior. Using these fitted parameters obtained from the DVS adsorption and desorption isotherms, the enthalpy associated with the hydration monolayer (Δ*H*_ads/des_) can be estimated by

9 $$\Delta H_{\mathrm{ads}/\mathrm{des}}=RT\ln\left(c_{i}\right)+k\Delta H_{\mathrm{cond}/\mathrm{vap}}$$

where *R* is the universal gas constant, *T* is the temperature (25 °C), *c_i_* is the model-specific sorption constant with = *c_i_* = *c*_G_ for the GAB model, and Δ*H*_cond/vap_ is the latent heat of condensation (−44 kJ/mol) and vaporization (+44 kJ/mol) for water.^48,49^ Following conventional thermodynamic sign conventions, enthalpies for adsorption and condensation are negative (Δ*H*_ads_ < 0, Δ*H*_cond_ < 0), whereas enthalpies for the reverse desorption and vaporization processes are positive (Δ*H*_des_ > 0, Δ*H*_vap_ > 0).^48,49^

#### 2.4.5 Differential Scanning Calorimetry (DSC) and Thermogravimetric Analysis (TGA)

Thermal analysis was conducted using a DSC-TGA Q600 SDT system (TA Instruments, New Castle, DE), enabling simultaneous DSC-TGA. Protein BCP samples (3 mg) were loaded into Tzero aluminum pans and equilibrated under controlled ambient conditions (*T* = 25 °C, 50% RH) for 30 min prior to hermetic sealing. The sealed pans were then scanned under N_2_ flow (50 mL/min), with temperature ramps from 25 to 250 °C at 10 °C/min.

### 2.5 Statistical Analysis

Descriptive statistics were computed for each appropriate experiment and reported as an average ± standard error across at least three independent batches or trials. Data was analyzed and compared using Excel (Version 2401, Microsoft, Redmond, WA). Groups with significant differences were determined using a Student’s *t*-test (when two groups were compared) and two-way ANOVA with *posthoc* Tukey Test (when three or more groups were compared). Differences were deemed statistically significant when the *p*-value <0.05 (*), *p* < 0.01 (**), *p* < 0.005 (***), or *p* < 0.001 (****).

## 3 Results and Discussion

### 3.1 Physicochemical Context of the L44A Mutation in Protein Diblock BCPs

The CE and C_L44A_E constructs differ by a single leucine-to-alanine substitution at position 44 within the C domain (Figure 1A). This substitution replaces the branched isobutyl side chain of leucine ∼164 Å^3^ with the significantly smaller methyl group of alanine 91 Å^3^, corresponding to an approximate 45% reduction (i.e., Δ*V*_vdw_ = 73 Å^3^) in steric side chain volume (Figure 1B).^50^ Mapping the primary sequence onto a helical wheel projection shows that residue 44 occupies the critical third “*a”* position of the heptad repeat (i.e., *abcdefg*) (Figure 1C). The L44A substitution alone is sufficient to trigger catastrophic unfolding, reducing α-helical content to below 50%.^51^

**Figure 1:**
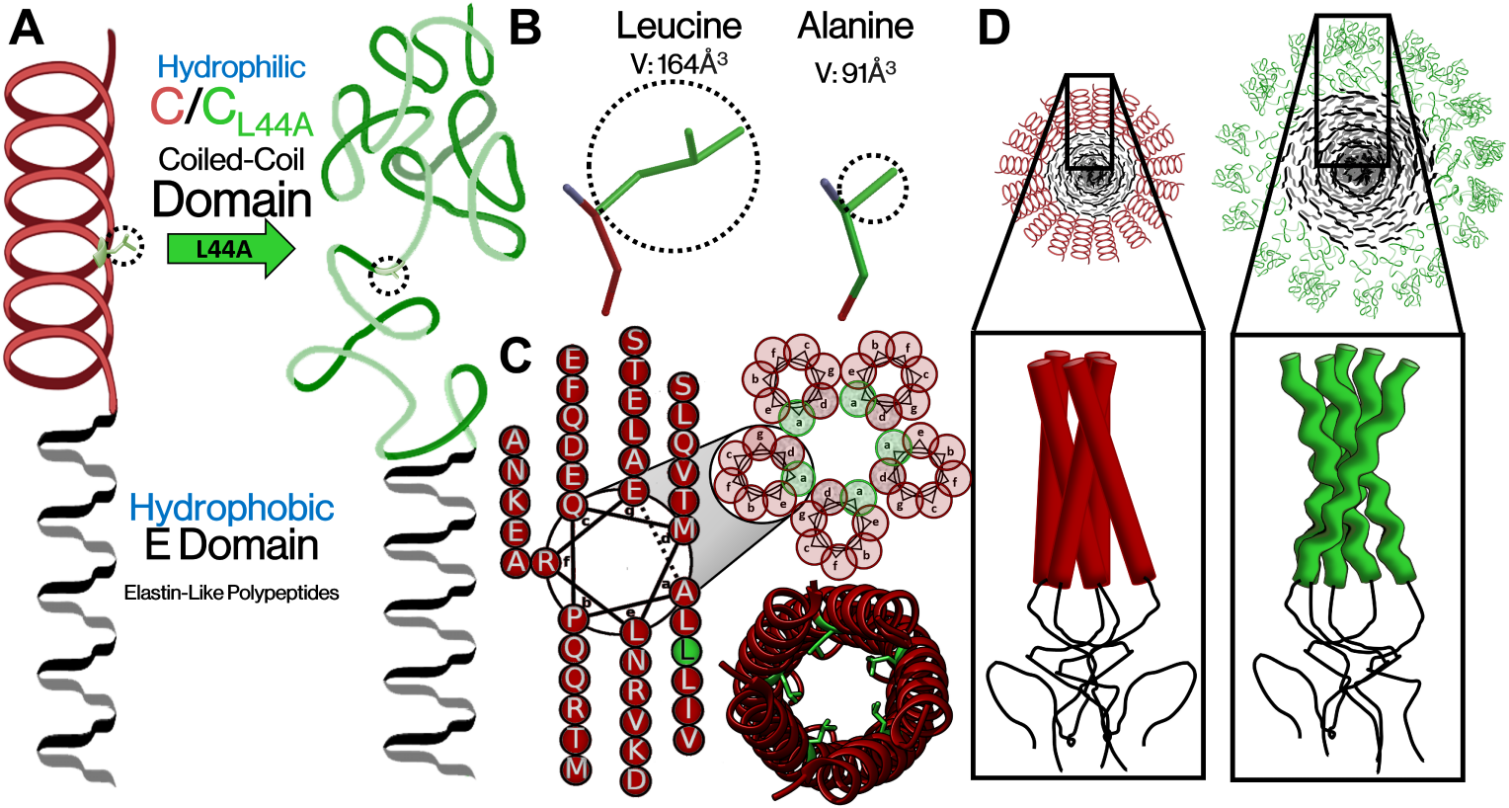
Hierarchical impact of the L44A mutation. (A) Schematic representation of CE and C_L44A_E diblock protein BCPs. (B) Comparison of leucine and alanine side-chain van der Waals volumes.^50^ (C) Helical wheel projection and cross-sectional view of the knobs-into-holes packing and leucine zipper motif within the canonical pentameric coiled-coil assembly of the C domain (PDB: 3V2P).^52^ (D) Supramolecular models of amphiphilic micelles featuring hydrophobic E domain cores and hydrophilic C or C_L44A_ shells. The bottom insets depict the rigid-rod representation of bundled coiled-coils in CE contrasted against the flexible, entangled chains of C_L44A_E.

Although the L44A mutation holds several structural and assembly ramifications, the global physicochemical properties of the two diblocks remain largely conserved as the primary sequences only differ by a single amino acid. As summarized in Table 1, both constructs display comparable molecular weights and intrinsic hydropathicity via their aliphatic indices (AI)^53^ and grand average of hydropathicity (GRAVY).^54^ As monoblocks, both C and C_L44A_ are hydrophilic with negative GRAVY values, with the values subtly differing by the slight reduction in hydrophobicity afforded by the L44A mutation. By contrast, the E domain remains hydrophobic, with an AI of 94.48 and a positive GRAVY value of 0.707. Thus, both diblocks possess amphiphilic character and assemble into core–shell micelles with hydrophobic, coacervated E-domain cores and hydrophilic coronas composed of C or C_L44A_ (Figure 1D). Although the E blocks are larger than the C/C_L44A_ blocks in both residue length (i.e., 125 vs 42) and theoretical specific volume (i.e., 12,700 Å^3^ vs 5,862/5,812 Å^3^),^55^ prior work has demonstrated the sensitivity of assembly behavior to the individual behavior of both blocks.^30^ Therefore, even the smaller C/C_L44A_ shell blocks are expected to exert a significant influence on supramolecular organization. In CE, the C domain adopts a relatively rigid, α-helical coiled-coil structure, behaving like anisotropic rods that promote directional packing at the micelle interface (Figure 1D, inset).^56–58^ Comparatively, disruption of helical structure and coiled-coil interactions in C_L44A_E is anticipated to yield a more flexible and conformationally heterogeneous corona. Nevertheless, these considerations are based on canonical dilute solution-phase behavior, and it remains unclear how these structural or assembly features manifest under desolvated or partially solvated conditions. Herein, we examine how this single-residue substitution hierarchically impacts secondary structure formation, supramolecular assembly, and hydration layer interactions across multiple hydration states.

**Table 1:**
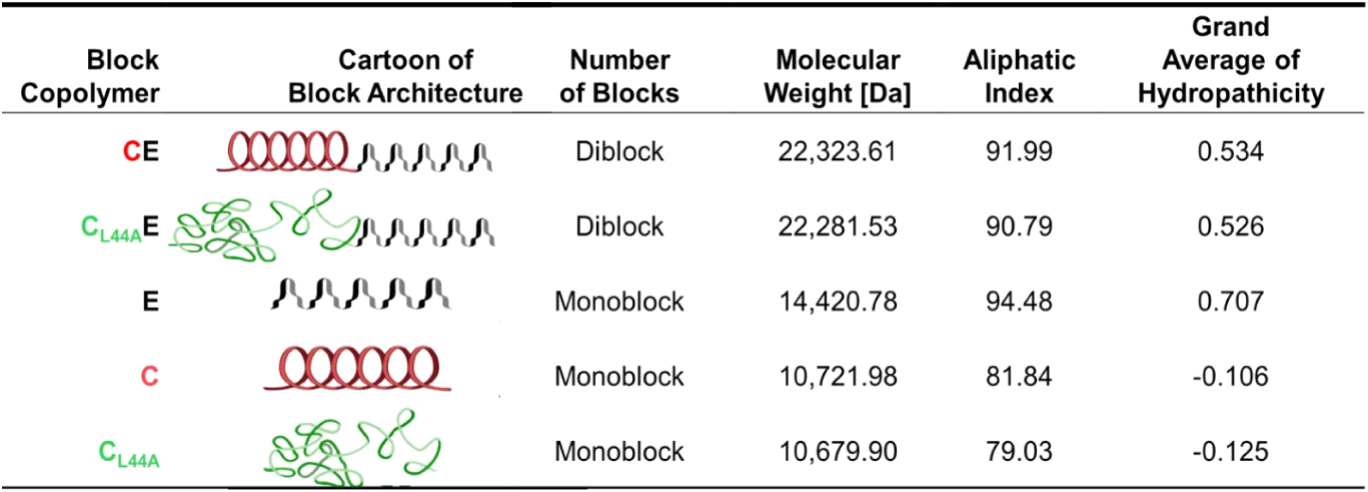
Architectures and Intrinsic Properties, Including Molecular Weight, Aliphatic index,^53^ and Grand Average of Hydropathicity,^54^ for Protein BCPs^a^

a Full sequences of amino acids are detailed in extended Table S1.

### 3.2 Expression, Purification, and Synthetic Verification of Protein BCPs

Using previously established protocols,^30–34,51^ we expressed and purified five protein constructs: three monoblock controls (C, C_L44A_, and E) and the two asymmetric diblock copolymers (CE and C_L44A_E). Across multiple expression batches, cultures generate 1.6–2.1 g of cell pellet per 400 mL culture, yielding 50–270 mg of lyophilized protein, depending on the construct. Purification by Ni-NTA affinity chromatography under denaturing conditions (i.e., 6 M urea) yields the target proteins, with SDS-PAGE showing dark bands around the expected molecular weights for all constructs (Figure S1). Both CE and C_L44A_E remain stable throughout expression, purification, and progressive refolding during stepwise urea dialysis, producing clear protein solutions at 4 °C without evidence of precipitation. To further verify molecular identity, MALDI-ToF MS performed on the purified constructs confirms the anticipated intact molecular weights via primary mass peak [M]^+^ within <0.05% of each expected theoretical intact mass (Figure 2, Tables 1, S2).

**Figure 2:**
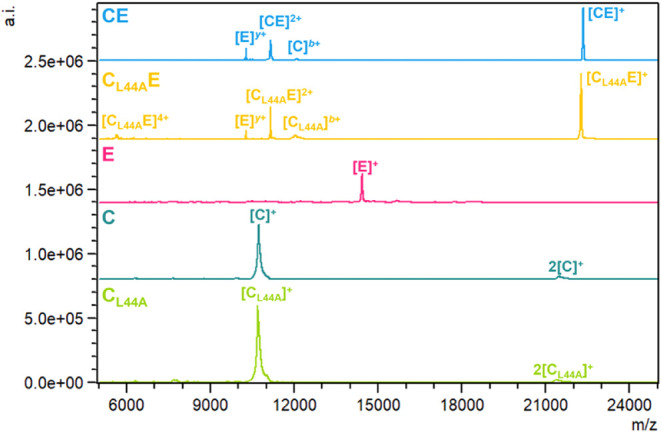
MALDI-ToF MS spectra, providing high-resolution confirmation of the expressed and purified protein BCPs constructs. Peaks are labeled with their assigned ions, with further quantification and detailed peak metrics provided in Table S2.

### 3.3 Characterization of Ensemble-Averaged Secondary Structure across Hydration States

As water acts as an active participant in stabilizing or destabilizing specific conformations, proteins can exhibit different local secondary structures depending on their hydration state.^59,60^ Capturing these structural shifts within the protein ensemble across physical phases remains challenging and often requires multiple complementary techniques. While high-resolution methods, such as solid-state nuclear magnetic resonance (ssNMR)^61^ and anomalous small-angle X-ray scattering (ASAXS),^62,63^ can provide specific structural insights, ensemble-averaged spectroscopic methods, like CD and FTIR, remain the most accessible and widely employed techniques.^60,64,65^ Accordingly, we use CD spectroscopy to probe protein secondary structure in the fully hydrated (i.e., wet) state and FTIR spectroscopy to examine the dehydrated (i.e., lyophilized) state.

CD spectra suggest that BCP secondary structures are not straightforward linear combinations of their constituent monomeric blocks; instead, one block appears to exert a dominant influence on the overall spectral profile (Figure 3A). C and CE display features characteristic of α-helical structure with the characteristic double minima at 208 and 222 nm, whereas C_L44A_ and C_L44A_E show reduced signal at 222 nm with a single minimum centered at 205 nm. The E monoblock exhibits a shifted minimum at 214 nm, consistent with β-sheet enrichment following spontaneous coacervation into a β-spiral above its phase transition temperature (*T*_t,E_ ∼ 25.0 °C).^31,35^ Detailed ellipticity values are provided in Table S3. To facilitate comparison across constructs, CD spectra were deconvoluted using the Beta Structure Selection (BeStSel) algorithm,^40^ which utilizes empirically derived reference spectra to assign secondary structure content (Figure 3B, Table 2). Constructs containing the C domain feature relatively higher ordered structural content, defined as the sum of α-helix and β-sheet, compared to those incorporating the C_L44A_ and E domains, which show a shift toward greater conformational disorder.

**Figure 3:**
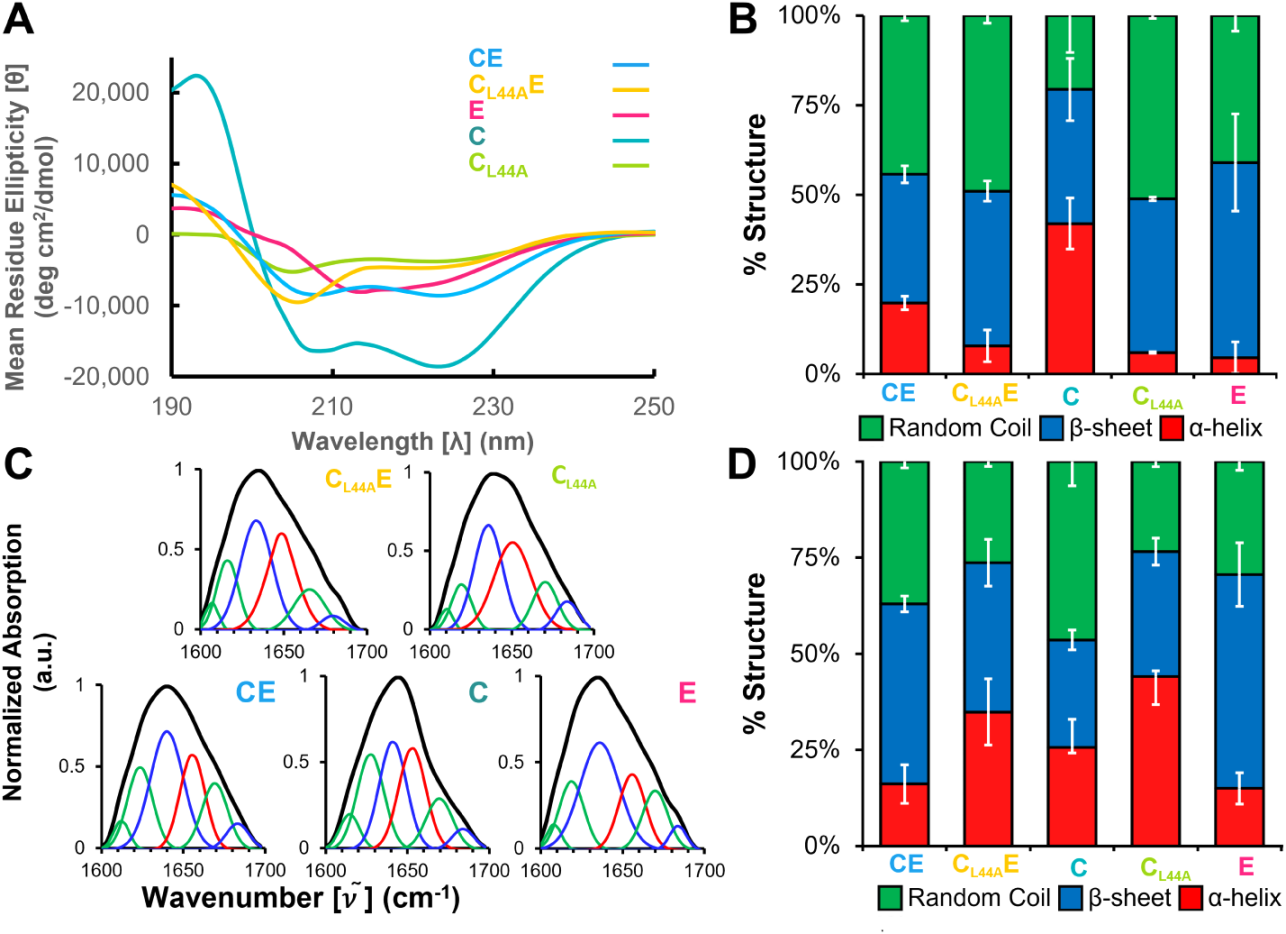
Secondary structural characterization of protein BCPs (A) Representative CD spectra of C_L44A_ (lime), C_L44A_E (gold), E (fuchsia), CE (sky blue), C (teal) in the hydrated (i.e., wet) state. (B) Secondary structure composition from CD spectra deconvolved into random coil (green), β-sheet (blue), and α-helix (red) using BeStSeL.^38^ Bars represent averages ± standard error from three independent replicates (C) Representative Amide I bands (ν̃ = 1600–1700 cm^–1^) from FTIR spectra of lyophilized protein with Gaussian deconvolution. (D) Quantitative secondary structure breakdown into random coil (green), β-sheet (blue), and α-helix (red) by percent.

**Table 2:** Comparison of Deconvolved Secondary Structure Content in the Hydrated (Wet) and Lyophilized (Dry) States Estimated from CD and FTIR, Respectively^a^

	Hydrated Secondary Structure from CD			Dry Secondary Structure from FTIR		
BCP	α-helix (%)	β-sheet (%)	Random coil (%)	α-helix (%)	β-sheet (%)	Random coil (%)
**CE**	19.8 ± 1.9	35.9 ± 2.4	44.3 ± 1.5	16.1 ± 5.0	46.8 ± 2.0	37.0 ± 1.6
**C**_**L44A**_ **E**	7.9 ± 4.4	43.1 ± 2.9	49.0 ± 2.1	34.8 ± 8.6	38.8 ± 6.0	26.3 ± 1.3
**C**	41.9 ± 7.1	37.4 ± 8.7	20.6 ± 10.3	25.6 ± 6.3	28.0 ± 2.6	46.4 ± 7.3
**C**_**L44A**_	6.0 ± 0.2	42.9 ± 0.5	51.2 ± 0.8	44.1 ± 1.5	32.5 ± 3.5	23.4 ± 1.3
**E**	4.6 ± 4.4	54.4 ± 13.5	41.0 ± 4.3	15.0 ± 4.1	55.6 ± 8.3	29.4 ± 2.3

a Values represent mean ± standard error.

To complement solution-state measurements, FTIR spectroscopy was performed on lyophilized BCPs (Figure 3C, 3D). The amide I region (ν̃ = 1600–1700 cm^–1^), arising primarily from backbone carbonyl (i.e., C=O) stretching vibrations, was fit to multicomponent Gaussians (Figure 3C), deconvoluted into subpeaks using second derivative analysis (Figure S2), and assigned to structural features in accordance with the Jackson model (Figure 3D).^41^ Relative to the solvated state, dehydration appears to promote changes in secondary structure content (Δ) in all BCPs (Figure 3, Table 2). Total structured content rose to 63.0 ± 5.4% (Δ^α+β^ = +7.3%) for CE and 73.7 ± 10.5% (Δ^α+β^ = +22.5%) for C_L44A_E. In CE, this increase was consistent with enrichment in β-sheet content (Δ^β^ = +10.9%) alongside a slight decrease in α-helical content (Δ^α^ = −3.6%), which matches previous reports of dehydration-induced protein restructuring via FTIR.^60^ By contrast, C_L44A_E exhibits a modest decrease in β-sheet content (Δ^β^ = −4.3%) and an increase in α-helical content (Δ^α^ = +27.0%). This trend was mirrored in the C_L44A_ monoblock (Δ^α^ = +38.1%), suggesting that dehydration shifts the conformational ensemble toward more ordered states.^66^

#### 3.3.1 Supramolecular Assembly and Mesoscale Morphology

Next, we examined the supramolecular assembly behavior of the protein diblock BCPs at the mesoscale using DLS, TEM, and SLS (Figure 4, Table 3). As anticipated for amphiphilic architectures, both diblocks assemble into micellar structures in solution, whereas the monoblocks do not.^32,34,67^ DLS measurements of hydrodynamic diameter (*D*_H_) in deionized water reveal that C_L44A_E forms monodisperse (i.e., PDI < 0.2) particles of 190.6 ± 31.9 nm that are more than 3.5 times the size of CE, which are 52.0 ± 2.0 nm (Figure 4A, Table 3). Proceeding to TEM, the dry particle diameters (*D*_D_) are significantly smaller compared to the hydrodynamic measurements; however, the overall size trends persist, with C_L44A_E forming larger particles of 41.6 ± 0.6 nm and CE forming smaller compact particles of 19.3 ± 0.3 nm (Figure 4B, C, Table 3). While discrepancies between *D*_H_ and *D*_D_ are often dismissed as drying artifacts, the extent of shrinkage in this context can provide insight into the degree of water incorporation within the assemblies.^68^ Here, the diameter of the C_L44A_E micelles shrinks by 4.6-fold upon drying, indicating a higher degree of swelling relative to CE (2.7-fold), which is further augmented in the corresponding swelling ratios (*Q*_v_) and protein volume fractions (ϕ) (Table 3).

**Figure 4:**
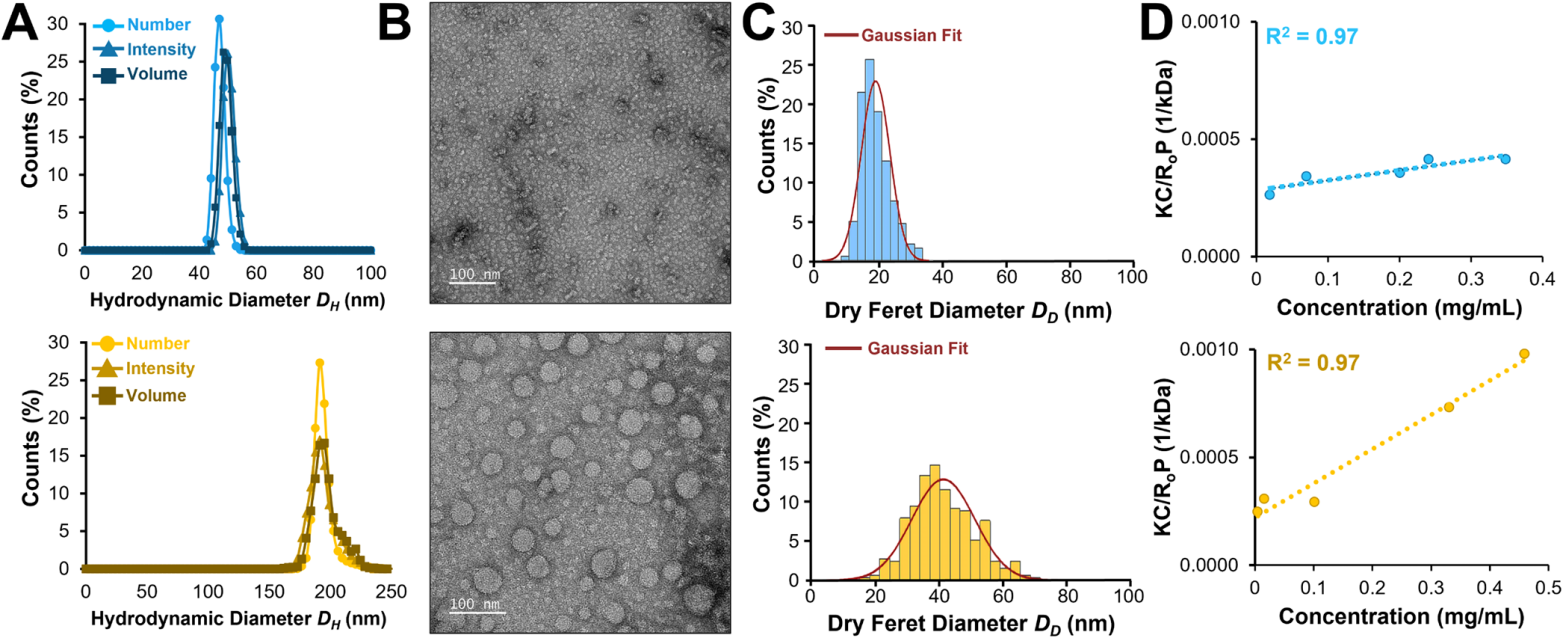
Supramolecular assembly characterization of CE (top, blue) and C_L44A_E (bottom, yellow) diblock protein BCPs. (A) DLS size distributions shown by number (circles), intensity (triangles), and volume (squares), illustrating the hydrodynamic diameters of solvated assemblies in deionized water. (B) Representative TEM micrographs of dried assemblies. Additional micrographs can be found in Figure S3. Scale bars: 100 nm (C) Histograms of TEM dry-state Feret diameters for *n* = 300 particles fitted to a normal Gaussian distribution. (D) Debye plots constructed from SLS measurements used to estimate protein–solvent interactions and M_w,assembly_ of each protein BCP assembly.

**Table 3:** Mesoscale Characterization of Diblock Micelle Assemblies Under Dilute and Dried Conditions

BCP	*D*_H_ (nm)	PDI_DLS_	*D*_D_ (nm)	PDI_TEM_	*Q*_v_	*ϕ*	*A*_2_ (mol·mL/g^2^)	*M*_w,assembly_ (kDa)	*N*_agg_ (monomers)
CE	52.0 ± 2.0	0.064	19.3 ± 0.3	0.072	19.6	0.051	1.00 × 10^–6^	3,546	159
C_L44A_E	190.6 ± 31.9	0.121	41.6 ± 0.6	0.062	96.2	0.010	1.59 × 10^–3^	3,257	146

SLS Debye plots indicate that CE and C_L44A_E form assemblies in solution with comparable *M*_w,assembly_ and *N*_agg_ (Figure 4D, Table 3), suggesting that the observed differences in size between constructs mainly arise from differences in chain packing and solvent uptake. CE possesses a higher packing density, forming tighter, less swollen micelles, whereas C_L44A_E forms more bloated assemblies. This is echoed by the second virial coefficient, which captures deviations in protein–solvent interactions.^69^ Through its contributions to osmotic pressure, *A*_2_ is directly proportional to (1/2 – χ), where χ is the Flory–Huggins polymer–solvent interaction parameter (see Supplementary Methods). In Flory–Huggins theory, χ determines the free energy of mixing and, therefore, the solvent chemical potential.^69^ Establishing this connection to thermodynamic mixing theory is important for hierarchically assembled WR protein materials, where swelling and contraction, and by extension, folding and unfolding, are all influenced by the chemical potential of water. Thus, as an experimentally accessible proxy for χ, *A*_2_ enables rational inference of how molecular and supramolecular-level interactions influence chemical potential gradients of water and, consequently, the thermodynamic driving force during swelling. Both CE and C_L44A_E exhibit positive *A*_2_ values, indicating overall favorable solvation conditions; however, CE displays a smaller *A*_2_ corresponding to a higher χ value, and C_L44A_E exhibits a substantially larger *A*_2_ value corresponding to a lower χ value. This supports the notion of stronger coiled-coil interactions at the CE micelle surface, which could occlude solvent infiltration (Figure 1D).

Compared to the previously reported triblock BCP systems, CE forms micelles of comparable size to CEC (i.e., *D*_H_: 58.9 ± 2.9 nm; *D*_D_: 28.5 ± 6.4 nm; *N*_agg_: 80.4), but with nearly twice (i.e., 1.96×) the monomer packing density.^34^ In contrast, C_L44A_E forms micelles that are 3.5 fold larger than its triblock analog, C_L44A_EC_L44A_ (i.e., *D*_H_: 53.9 ± 1.5 nm; *D*_D_: 30.6 ± 5.3 nm; *N*_agg_: 31.2), while also displaying a 4.7 fold increase in monomer packing.^34^ These differences in assembly likely arise from the amphiphilic architectural constraints of the triblock, which favor configurations that maintain solvent exposure at both of its hydrophilic C/C_L44A_ termini, thereby limiting its packing efficiency. Therefore, an amphiphilic BCP architecture is necessary to drive micellization, but the hydropathy and conformational flexibility of the constituent end blocks ultimately dictate the size and packing density of the resultant micelles.^70^

#### 3.3.2 Morphological Comparison of Drop-Casted Protein BCP Films

To investigate how each diblock assembly translates into solid-state films, we casted the reconstituted lyophilized protein BCPs assemblies onto PI substrates and visualized them under ESEM (Figure 5). ESEM probes how these assemblies organize, providing insight into the macroscopic arrangement and surface topology of the cast films. CE films form relatively smooth, featureless surfaces, matching the smooth, densely packed structures observed for previously reported triblock films.^33^ On the other hand, C_L44A_E film displays highly porous microstructures, rife with interconnected surface irregularities and microscale cavities. Following one sorption cycle, the C_L44A_E films maintain their structural integrity, undergoing reversible swelling and contraction without visually apparent changes, whereas CE films develop microtears and fractures (Figure 5).

**Figure 5:**
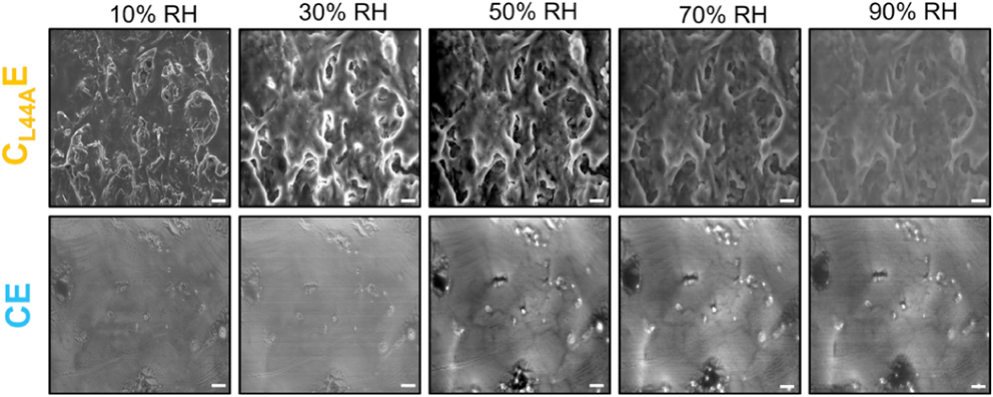
Representative ESEM micrographs showing C_L44A_E and CE film surface morphologies from the top across one cycle of 10% to 90% RH. *Scale bars*: 10 μm.

We attribute this juxtaposition in film morphology to the preorganization of micellar assemblies. During film formation, supramolecular micelles are drop-casted onto the PI substrate, where their packing tendencies may be predetermined by the structural characteristics of their constituent domains. CE assemblies, incorporating the more rigid and directionally ordered C block, pack more efficiently, resulting in smoother films with visually smaller void spaces.^64,71^ Comparatively, C_L44A_E, with its larger micelles, packs less efficiently, generating films with larger gaps and a macroscopically porous network.^72^ Such structures are reminiscent of coalesced micellar networks reported for synthetic, nonprotein BCP systems, like Pluronic, where semi-bald corona coverage of micelles facilitates intermicellar bridging and the formation of porous, percolated networks.^73–75^

### 3.4 Probing Hydration Layer Interactions with Protein BCP Films

To elucidate how hydration layers interact with CE and C_L44A_E assemblies, we employed three complementary techniques: (i) DVS to measure the overall extent of water uptake at equilibrium and estimate sorption energetics as a function of RH; (ii) FTIR to provide insight into the chemical H-bonding environments of nanoconfined water, and (iii) DSC-TGA to examine the stability of each construct to thermal perturbation (Figure 6). Compared to CE, which shows more steady water influx reaching maximum sorption of ∼122%, the C_L44A_E DVS isotherm exhibits lower initial water uptake, but a sharp, nonlinear increase in mass around 70% RH, reaching ∼250% maximum water uptake at 95% RH (Figure 6A). Notably, the magnitude of water uptake for both diblocks exceeds that of the monoblocks and previously reported triblocks,^33^ which exhibited less than ∼50% maximum mass change over a similar RH range (Figure S4). This enhanced uptake in C_L44A_E could be attributed, at least in part, to increased solvent-accessible free volume (i.e., 1 – ϕ) within the packed micelle film (Figure S5). The larger pores of C_L44A_E are less effective at stabilizing initial adsorption, but could facilitate greater water incorporation once the multilayer reaches saturation. Isotherm fitting and calculation of the enthalpy associated with the initial hydration layer (Δ*H*_ads_) further clarifies the hydration energetics of each BCP system (Figure 6A). Below ∼70% RH, C_L44A_E displays a more negative Δ*H*_ads_ than CE (Δ*H*_ads,CE_ = −43.51 kJ/mol; Δ*H*_ads,CL44AE_ = −62.62 kJ/mol), indicating stronger initial protein–solvent interactions and a more stabilized first hydration layer. However, when analyzing the desorption isotherm above ∼70% RH, the fitted enthalpy is greater for CE (Δ*H*_des,CE_ = +44.29 kJ/mol) than for C_L44A_E (Δ*H*_des,CL44AE_ = +35.32 kJ/mol), suggesting a higher energetic penalty for removing water from the nanoconfined and cooperative environment of CE.

**Figure 6:**
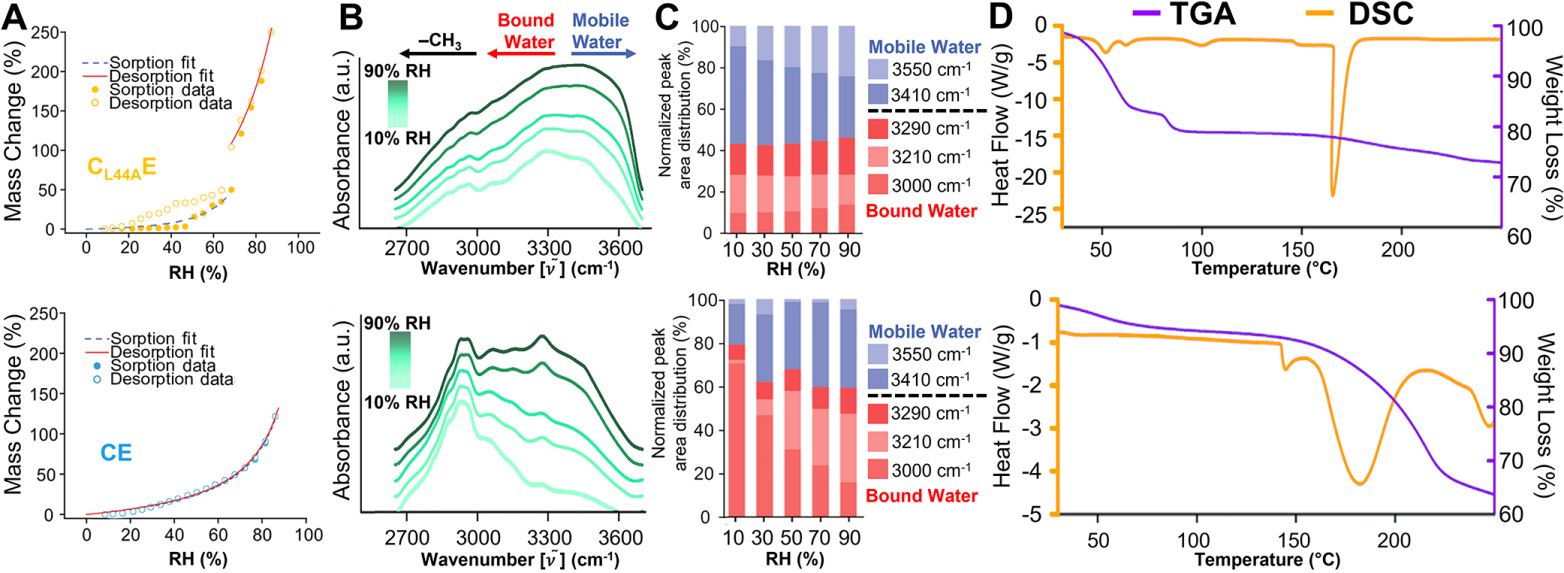
Characterization of the hydration layer interactions in C_L44A_E (gold, top) and CE (sky blue, bottom). (A) DVS equilibrium water sorption–desorption isotherms with GAB model fits. (B) Normalized O–H stretching region (2700–3700 cm^–1^) of the FTIR spectra showing evolution of H-bonding networks in diblock protein BCPs under increasing RH. Spectra have been vertically offset for clarity. (C) Relative proportions of bound and mobile water determined via second-derivative peak deconvolution (see Figures S6, S7 for details) at 10%, 30%, 50%, 70%, and 90% RH. (D) DSC-TGA thermograms for C_L44A_E and CE.

To further resolve how hydration layers evolve within CE and C_L44A_E, we analyzed the O–H stretching region (2700–3700 cm^–1^) of the FTIR spectra under varying RH. Normalizing to the total integrated peak area, we deconvoluted the band into five subpeaks using second derivative analysis following the Podbevšek model (Figures 6B, C, S6).^42^ In general, stronger H-bonding lengthens the O–H bond, lowering its vibrational frequency and shifting absorbance to lower wavenumbers; similarly, weaker H-bonding leads to shorter O–H bonds with higher vibrational frequencies, resulting in absorbances at higher wavenumbers.^76,77^ Although the assignment of specific mobile and bound spectral regions is model-dependent, the designations were applied consistently to enable direct comparisons between CE and C_L44A_E.^78^ Across all RH levels, CE maintains higher bound-to-mobile water ratios than C_L44A_E, confirming differences in water partitioning in each BCP (Figure 6C). To complement this normalized partition analysis and account for increasing water uptake, Figure S7 shows the bound and mobile water contributions relative to the 10% RH baseline. In CE, the bound water fraction decreases progressively with increasing RH, indicating that newly absorbed water is incorporated mostly as mobile water, thereby reducing the relative contribution of bound water despite an increase in its absolute amount. By contrast, C_L44A_E maintains a nearly constant bound-to-mobile fraction despite greater water uptake, suggesting more dynamic exchange between mobile and bound water during RH cycling. Similar fluxional water lability has been reported to facilitate plasticization and structural rearrangement in other protein-based biomaterials.^7,27,71,79^

Complementing the DVS and spectroscopic hydration analyses, DSC-TGA were employed to probe the water retention and desorption process through thermal perturbations. Low temperature mass loss and thermal events in DSC-TGA generally reflect desorption of mobile water; however, in hierarchical protein assemblies like our BCPs, these processes overlap with several different protein shape-changing events (e.g., local domain unfolding, coacervation of the E block, entangled chain liberation, micelle collapse, etc.) that lower energetic barriers for bound-to-mobile water conversion and solvent release. Nevertheless, the DSC-TGA thermograms can provide qualitative insights into the conformational heterogeneity and cooperativity of each system.

CE exhibits a broad, smooth DSC thermal trace consistent with a more cooperative thermal response and a narrower conformational ensemble. Conversely, C_L44A_E shows a more irregular thermogram with multiple thermal events between 25–180 °C, indicative of a broader range of accessible structural and assembled states (Figures 6D, S8). Coupling TGA to these transitions, C_L44A_E loses ∼21.8 wt %, and CE only loses ∼7.1 wt % below 180 °C, consistent with the liberation of mobile water from the protein scaffold.^80^ At temperatures above 180 °C, CE experiences a ∼22.1% greater mass loss compared to C_L44A_E, consistent with cooperative coiled-coil denaturation^81^ followed by the onset of protein thermal decomposition around ∼250 °C for CE and ∼300 °C for C_L44A_E (Figures 6D, S9).^82,83^ Thus, while the L44A mutation undoubtedly impacts the molecular and supramolecular-level organization of protein BCPs, it also fundamentally reconfigures the dynamic behavior of the hydration shell surrounding the protein BCP scaffold.^84,85^

### 3.5 Macroscale WR Performance of Protein BCP/PI Films

Upon modulation of RH, both BCP/PI bilayer actuators exhibited pronounced macroscopic changes in curvature (i.e., out-of-plane bending), resulting from a mismatch in anisotropic, in-plane stress and strain generated from simultaneous hydration-induced swelling and WR structural rearrangements within the top BCP active layer and the passive PI layer (Figures 7A, B, S10).^86–88^ At RH 10%, the C_L44A_E bilayers underwent marked bending, whereas CE bilayers remained largely planar (Figure 7A, B). The C_L44A_E diblock achieved the highest curvature at 2.4 mm^–1^ and corresponding *U*_C_L44A_E_ of 2,043.1 ± 221.6 kJ/m^3^, representing a 2.7-fold increase over the previously top-performing C_L44A_EC_L44A_ triblock at 751.7 ± 66.9 kJ/m^3^ (*p* = 0.0051) (Figure 7C).^33^ The CE diblock followed with a lower energy density of 481.4 ± 45.6 kJ/m^3^, significantly below C_L44A_EC_L44A_ (*p* = 0.0001). The CEC triblock also demonstrated reduced curvature (Γ_CEC_ = 0.45 ± 0.1 mm^–1^) and performance (*U*_CEC_ = 100.3 ± 9.1 kJ/m^3^), underperforming both the CE diblock (*p* = 0.0022) and its C_L44A_EC_L44A_ counterpart (*p* = 0.0006).^33^ All monoblocks (i.e., C_L44A_, C, E) displayed minimal curvatures (Γ_CL44A_ = 0.44 ± 0.10 mm^–1^, Γ_C_ = 0.37 ± 0.10 mm^–1^, Γ_E_ = 0.27 ± 0.10 mm^–1^) and correspondingly low *U* values (*U*_CL44A_ = 72.2 ± 6.7 kJ/m^3^, *U*_C_ = 42.4 ± 3.5 kJ/m^3^, *U*_E_ = 27.5 ± 1.9 kJ/m^3^). When stratifying by domain type, constructs containing the C_L44A_ domain significantly outperform those containing the wild-type C domain (*p* = 0.0002). Moreover, when stratifying by the number of blocks, diblocks were found to significantly outperform both triblocks (*p* = 0.0446) and monoblocks (*p* = 0.0011), reinforcing the importance of multidomain, amphiphilic architecture for WR behavior.

**Figure 7:**
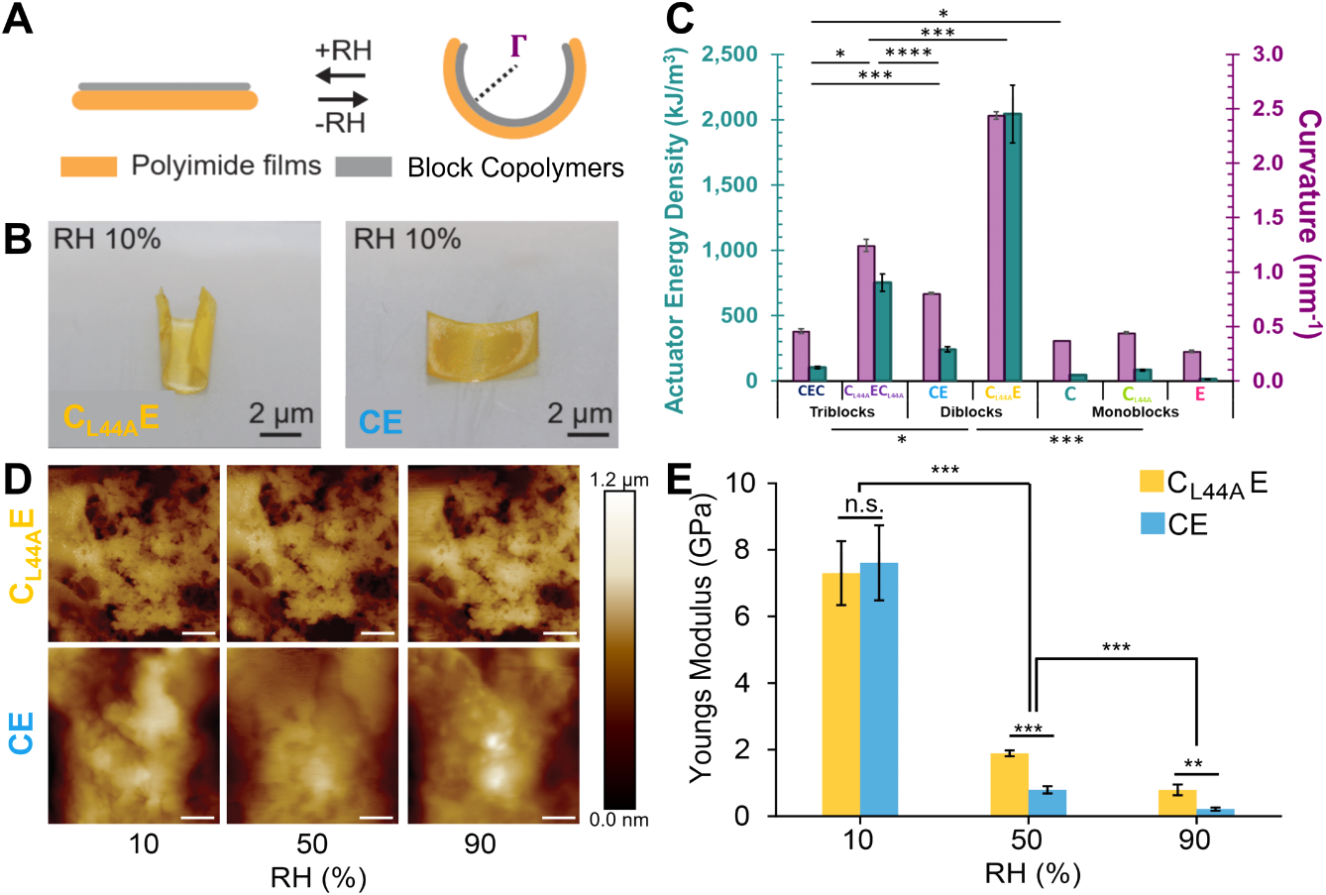
(A) Conceptual schematic depicting the BCP/PI bilayer actuators. (B) Representative images of C_L44A_E and CE bilayer films under 10% RH. *Scale bar*: 2 μm. (C) Quantitative comparison of film curvatures and calculated actuator energy density across various WR materials. Data for CEC and C_L44A_EC_L44A_ adapted and reproduced from [33] for comparison. (D) AFM micrographs of BCP/PI films used to map topological changes at 10%, 50%, and 90% RH. *Scale bars:* 2 μm (E) Calculated Young’s moduli values from AFM nanoindentation experiments for C_L44A_E (gold) and CE (sky blue). *p*-value <0.05 (*), *p* < 0.01 (**), *p* < 0.005 (***), or *p* < 0.001 (****).

AFM tomography monitors the film topography and spatial variations in the diblock BCP active layer across RH cycling (Figure 7D). With increasing RH, CE demonstrates more pronounced changes in surface morphology, with features that smooth and reorganize across the surface. In contrast, C_L44A_E maintains a more constant, textured topography, suggesting that its structural changes are more localized, possibly to the C_L44A_ block, and less cooperative. Nanoindentation measurements provided further insight into the RH-dependent mechanical stiffness of each BCP. At RH 10%, both diblocks demonstrate comparable baseline stiffness (*p* = 0.8535), with similar Young’s moduli of 7.3 ± 1 GPa and 7.6 ± 1.1 GPa for C_L44A_E and CE, respectively (Figure 7E, S11). These values are comparable to previously reported moduli for ultrathin regenerated silk fibroin films in the range of 6–8 GPa.^89^ As RH increased to 50%, both samples demonstrate a significant decrease in stiffness (*p* = 0.0604), with C_L44A_E softening to 1.9 ± 0.1 GPa and CE to 0.8 ± 0.1 GPa. At 90% RH, this trend is more pronounced with C_L44A_E maintaining a modulus of 0.8 ± 0.2 GPa, significantly higher (*p* = 0.0039) than the CE modulus of 0.2 ± 0.1 GPa (Figure 7E).

Although water acts as a plasticizer in both systems, CE softens more readily under humid conditions, whereas C_L44A_E retains greater stiffness despite increased water uptake. Akin to other elastomeric (e.g., elastin,^90–92^ resilin,^92^ silk fibroin,^79^ etc.) and cartilage-derived collagen proteins,^93^ the BCPs contain differing ratios of disordered to ordered domains associated with each construct’s conformational ensemble. As rehydration augments the conformational space available to each BCP, C_L44A_E gains access to a broader range of microstates than CE, allowing hydration-induced shape change to proceed through a larger number of local structural states that provide additional pathways toward viscoelastic relaxation and energy dissipation.^94–97^ This behavior enables C_L44A_E to structurally adapt to preserve protein–water interactions at elevated RH (Figure 6C), which can explain the retained stiffness and sorption hysteresis observed in the C_L44A_E AFM nanoindentation (Figure 7E) and DVS isotherms (Figure 6A), respectively. This interpretation is further bolstered by the feature-rich C_L44A_E DSC-thermogram and the cooperative thermal response of CE, which are consistent with greater conformational heterogeneity in C_L44A_E compared to CE. Notably, the previously characterized triblocks (i.e., C_L44A_EC_L44A_ and CEC) also parallel this behavior of the diblock, with C_L44A_EC_L44A_ maintaining greater stiffness at 90% RH relative to CEC.^33^ As a result, macroscopic actuation in these BCP systems stems from structural and supramolecular assembly rearrangements constrained by the critical hydration layer.

## 4 Conclusion

This work demonstrates that a single, sequence-level mutation is sufficient to reprogram structure, self-assembly, and WR actuation across hierarchical length scales in protein diblock BCPs. The L44A mutation alters secondary structure evolution during desolvation, promoting broader structural ensembles in C_L44A_E that diverge from the traditional, cooperative folding-unfolding transitions observed for the wild-type CE. Such sensitivity of protein structure to minimal sequence perturbations is well-established, as even single-residue changes are known to shift folding pathways, stabilize metastable conformations, and reshape functional outcomes.^29,98^ These sequence-encoded structural differences propagate into supramolecular assembly, where C_L44A_E forms larger, less densely packed micelles compared to the compact, efficiently packed CE assemblies. Similar sequence-level control of assembly morphology has been reported in coiled-coil systems and peptide-based biomaterials, where single amino acid substitutions redirect aggregation, packing efficiency, and mechanical response.^99,100^ Here, these effects extend across length scales, manifesting in solid-state films with dramatically different porosity, water uptake, and humidity-dependent mechanical behavior. While C_L44A_E exhibits substantially greater water uptake, this hydration is predominantly comprised of mobile, bulk-like water, whereas CE supports more tightly confined and cooperative hydration. By linking the second virial coefficient to the Flory–Huggins interaction parameter, these differences can be interpreted in terms of the chemical potential of water, establishing a thermodynamic framework that connects molecular interactions to swelling behavior. Within this framework, WR actuation emerges from hydration-driven protein structural rearrangements. The combination of enhanced water uptake, preserved stiffness, and distributed hydration in C_L44A_E enables greater internal stress generation and retention, resulting in its exceptionally high actuation energy density of 2,043 kJ/m^3^. This value represents the highest reported for this class of protein BCP actuators and underscores how single-point mutations can amplify into large functional gains through coupled structural and assembly mechanisms. By connecting mutation-driven folding changes to supramolecular packing, hydration thermodynamics, and macroscopic actuation, this work demonstrates how we can harness protein sequence–structure–assembly relationships for autonomous, high-performing energy-harvesting biomaterials.

## Supplementary Material







## Notes

Experimental methods, figures, and extended tables, including FTIR second derivative analysis, MALDI-ToF-MS peak assignments, additional TEM micrographs, equilibrium water sorption isotherms, force–separation curves, and expanded DSC-TGA thermograms for C, C_L44A_, and E monoblocks and BCP constructs (PDF)
Real-time actuation motion of the diblock protein BCP film (MOV)
Real-time actuation motion of the diblock protein PI film (MOV)

## Abbreviations

WR: water-responsive
RH: relative humidity
H-bond: hydrogen bond
BCP: block copolymer
SAD: self-assembly domain
C: coiled-coil domain
COMP: Cartilage Oligomeric Matrix Protein
E: elastin-like polypeptide domain
C_L44A_: leucine-to-alanine mutated C domain
*E. coli*: *Escherichia coli*
AF-IQ: phenylalanine auxotrophic BL21­(DE3) *E. coli*
AMP: ampicillin
CAM: chloramphenicol
Ni-NTA: nickel-nitrotriacetic acid
NaOH: sodium hydroxide
IPTG: isopropyl β-d-1-thiogalactopyranoside
Tris HCl: Tris­(hydroxymethyl)­aminomethanehydrochloride
SDS: sodium dodecyl sulfate
CAN: acetonitrile
BCA: bicinchoninic acid
MWCO: molecular weight cut off
VitB: thiamine hydrochloride
CHCA: α-cyano-4-hydroxycinnamic acid
SA: sinapinic acid
TFA: trifluoroacetic acid
HCl: hydrochloric acid
PI: polyimide
MALDI-ToF MS: matrix-assisted laser desorption ionization-time-of-flight mass spectrometry
S/N: signal-to-noise
fwhm: full width at half-maximum
CD: circular dichroism
FTIR: Fourier-transform infrared
DLS: dynamic light scattering
SLS: static light scattering
TEM: transmission electron microscopy
ESEM: environmental scanning electron microscopy
AFM: atomic force microscope
DVS: dynamic vapor sorption
GAB: Guggenheim–Anderson–de Boer
DSC: differential scanning calorimetry
TGA: thermogravimetric analysis
SDS-PAGE: sodium dodecyl sulfate polyacrylamide gel electrophoresis
*T*_t_: transition temperature
*V*_vdw_: van der Waal’s Volume
α: α-helix
β: β-sheet
*R*: random coil
λ: wavelength
*ν̃*: wavenumber
Δ: change
*D*_H_: DLS-derived hydrodynamic diameter
*D*_D_: TEM-derived dry Feret Diameter
PDI: polydispersity index
*Q*_v_: volumetric swelling ratio
ϕ: protein volume fraction
χ: Flory–Huggins interaction parameter
*M*_w,assembly_: molecular weight of assembly
*N*_agg_: aggregation number
AI: aliphatic index
GRAVY: grand average of hydropathicity
Γ: curvature
*U*: actuation energy density
Δ*H*_ads/des_: energy associated with adsorption/desorption of the initial hydration monolayer
*E*: Young’s modulus
