# Supplementary material for: Single-Residue Mutation Switch Reconfigures the Hierarchical Structure and Assembly of Amphiphilic Protein Block Copolymers for Hydration Layer-Dominated Water-Responsive Actuation
Source: Biochemistry. 2026 Jun 22;65(13):2165–79. doi: 10.1021/acs.biochem.6c00038 (PMC13348036; doi:10.1021/acs.biochem.6c00038)
Supplement: Supplementary file 1 [file bi6c00038_si_001.pdf]

Supporting Information

Single-Residue Mutation Switch Reconfigures Hierarchical Structure and Assembly of Amphiphilic Protein Block Copolymers for Hydration Layer Dominated Water-Responsive Actuation

Jonathan W. Sun,[1,2] Chengyu Sun,[3,5] Seungri Kim,[3,5] Andrew L. Wang,[1,4] Isabella Huang,[1] Nada Haq-Siddiqi,[1] Zara Hedaya,[1] Raymond S. Tu,[3] Xi Chen,[3,5,6,*] Jin Kim Montclare[1,2,7,8,9,*]

[1]Department of Chemical and Biomolecular Engineering, New York University (NYU) Tandon School of Engineering, Brooklyn, NY 11201, USA
[2]Department of Chemistry, New York University, New York, NY 10003, USA
[3]Department of Chemical Engineering, City College of New York, New York, NY 10031, USA
[4]Department of Biomedical Engineering, State University of New York (SUNY) Downstate Health Sciences University, Brooklyn, NY 11203, USA
[5]Advanced Science Research Center (ASRC) at the Graduate Center, City University of New York, New York, NY 10031, USA
[6]PhD Programs in Chemistry and Physics at the Graduate Center, City University of New York, New York, NY 10016, USA
[7]Department of Biomedical Engineering, NYU Tandon School of Engineering, Brooklyn, NY 11201, USA
[8]Department of Radiology, NYU Langone School of Medicine, New York, NY 10016, USA
[9]Department of Biomaterials, NYU College of Dentistry, New York, NY, 10010, USA

*Corresponding authors: Jin Kim Montclare, PhD. Email: montclare@nyu.edu
Xi Chen, PhD. Email: xchen@gc.cuny.edu

This PDF includes:

Supplementary Methods
- Protein Block Copolymer Expression
- Protein Block Copolymer Purification
- Additional Notes on MALDI-ToF/ToF-MS Peak Assignments
- Actuator Energy Density Geometric Term Expansion
- Sensitivity Analysis of Young's Modulus Dependence
- Derivation of A_2 Proportionality to Flory-Huggins Interaction Parameter

Supplementary Figures (S1-S12)
- **Figure S1.** SDS-PAGE purification gels for monoblock and diblock protein BCPs
- **Figure S2.** Second-derivative FTIR analysis for protein BCPs
- **Figure S3.** Additional TEM Micrographs for Feret diameter quantification
- **Figure S4.** DVS Sorption-Desorption Isotherms for all protein BCPs
- **Figure S5.** Correlative analysis for water sorption
- **Figure S6.** Deconvolutions and second-derivative FTIR analysis for hydration layers
- **Figure S7.** Bound and water molecule normalized to DVS water uptake
- **Figure S8.** Zoomed DSC trace showing thermal transitions for CE and $C_{L44A}E$
- **Figure S9.** Extended TGA thermogram showing disintegration events for CE and $C_{L44A}E$
- **Figure S10.** Reversible humidity cycling of diblock protein BCP/PI actuator films
- **Figure S11.** Force-separation curves from AFM nanoindentation
- **Figure S12.** Sensitivity analysis of Young's modulus impact on actuator energy density

Supplementary Tables (S1-S3)
- **Table S1.** Amino acid sequences for protein BCPs discussed in this work
- **Table S2.** MALDI-ToF/ToF-MS peak parameters and assignments
- **Table S3.** Notable extrema in CD spectra for protein BCPs discussed in this work

Supplementary References

Supplementary Methods

Protein Block Copolymer Expression

Cloning of the constructs was carried out using the techniques as described in previous papers.[1-3] Protein block copolymers were expressed and purified as previously described.[2-5] AF-IQ *E. coli* cells were transformed with previously cloned pQE-30 expression vectors encoding the desired protein block copolymer constructs (i.e. C, C_{L44A}, E, CE, and $C_{L44A}E$).[2-5] Transformants were selected by plating on tryptic soy agar containing 200 µg/mL AMP and 34 µg/mL CAM to select for successful transformants. Isolated colonies were picked and used to inoculated 10 mL of complete M9 minimal media containing: Na_2HPO_4 (0.5 M), KH_2PO_4 (0.22 M), NaCl (0.08 M), NH_4Cl (0.18 M), all 20 of the canonical amino acids (1 mg/mL each), $MgSO_4$ (1 mM), $CaCl_2$ (0.1 mM), 0.2% (w/v) D-(+)-glucose solution, 0.2% (v/v) trace metal solution ($CaCl_2$ (40 mM), $MnCl_2$ (20 mM), $CoCl_2$ (4 mM), $ZnSO_4$ (20 mM), $CuCl_2$ (4 mM), $NiCl_2$ (4 mM), Na_2SeO_3 (4 mM), H_3BO_4 (4 mM), Na_2MoO_4 (4 mM), and $FeCl_3$ (40 mM)), AMP (200 µg/mL), CAM (34 µg/mL), and VitB (35 µg/mL). For protein expression, 400 mL of complete M9 media prepared as described above was used with a 50/52 autoinduction mixture consisting of 0.5% (w/v) glycerol, 0.05% (w/v) D-(+)-glucose, and 0.2% (w/v) α-D-lactose monohydrate replacing the 0.2% (w/v) D-(+)-glucose solution [6]. Expression cultures were grown at 37 °C at 350 rpm for ~8 h, then harvested by centrifugation at 4,000 rpm for 10 min at 4 °C in an Avanti JXN-26 centrifuge (Beckman Coulter, Brea, CA).

Protein Block Copolymer Purification

For purification, pellets were resuspended in Buffer A (50 mM Na_2HPO_4, 6 M urea, 20 mM imidazole, pH 8) and lysed by sonication using a Q500 Sonicator (QSonica LLC, Newton, CT) for 2.5 min, 5 ON, 30 OFF, at 60% amplitude on ice. Clarified lysates were incubated overnight with Ni-NTA resin at 4°C on a Labquake™ tube shaker/rotator (Barnstead Thermolyne Corporation, Dubuque, IA) and purified by gravity-flow immobilized metal affinity chromatography at 4 °C. Resin was washed with Buffer A to remove non-specifically bound proteins, and proteins of interest were eluted with an increasing gradient of Buffer B (50 mM Na_2HPO_4, 6 M urea, 500 mM imidazole, pH 8). Pure elutions identified by SDS polyacrylamide gel electrophoresis (SDS-PAGE) were pooled and dialyzed (3.5 or 10 kDa MWCO as appropriate) stepwise against 50 mM Na_2HPO_4 with decreasing concentrations of urea (4 M, 2 M, 1 M, 0.5 M, 0.25 M), followed by pure deionized water to remove salts. Samples were concentrated using centrifugal filters (3.5 or 10 kDa MWCO as appropriate, 2,500×g, 4 °C), quantified by BCA assay using a BSA standard curve, flash-frozen, and lyophilized on a FreeZone Freeze Dryer System (Labconco, Kansas City, MO) to yield purified protein as dry powders.

Additional Notes on MALDI ToF/ToF-MS Peak Assignments

Some evidence of coiled-coil dimerization was observed for C and C_{L44A} at two times the primary mass peak $2[M]^+$. For CE and $C_{L44A}E$, the observed masses precisely matched the theoretical mass of the fused domains ($[M]^+$), the double-charged species ($[M]^{2+}$) at roughly half the intact mass, and the fragmented E-domain y ion and C-domain b ion, confirming successful synthesis of each diblock BCP (**Fig. 1, Table S2**). Protein backbone cleavage at the proline N-terminus and aspartic acid C-terminus, well-established hotspots for peptide bond scission during ionization,[7, 8] yielded complementary b_n (charge retained on N-terminal fragment) and y_m fragments (charge retained on the C-terminal fragment), corresponding to the predicted masses of the C/C_{L44A} and the E domains, respectively.

Actuator Energy Density Geometric Term Expansion
The actuation energy density (*U*) was calculated using the elastic bilayer bending model in **Eq. 5**. The area of moment of inertia for the BCP layer was calculated as:

$$I_1 = \frac{bt^3}{12} \quad \textbf{(Eq. S1)}$$

The area moment of inertia of the substrate layer is given by:

$$I_2 = \frac{bt_1^3}{12} + bt_1\left(\frac{t_1}{2} - H\right)^2 \quad \textbf{(Eq. S2)}$$

where t_1 is the thickness of the polyimide substrate and H is the location of the neutral axis of the substrate, determined from the Stoney equation:[9]

$$H = t_1 - \frac{E_1^2 t^4 + E_1 E_2 t t_1^2 (3t + 4t_1)}{6E_1 E_2 t t_1 (t + t_1)} \quad \textbf{(Eq. S3)}$$

Sensitivity Analysis of Young's Modulus Dependence
Because the Young's Modulus of the actuators varies with RH, the use of a single E_1 value in the aforementioned energy density calculation may, in principle, introduce compounding error. To evaluate the extent of this effect, we performed a sensitivity analysis in which E_1 was systematically isolated and varied over the experimentally determined range for $C_{L44A}E$ (i.e., 0.8–7.3 GPa), while all geometric parameters and substrate properties were held constant. Normalizing the resulting values to the reported energy density value (i.e. 2,043.1 kJ/m^3) corresponding to E_1 = 7.3 GPa, *U* shows near-negligible dependence on E_1 with the lowest value differing by only 5.5% (**Fig. S12**). Thus, the contribution of geometric factors dominates the overall density in these bilayer films, and using a single modulus value at 10% RH provides a consistent and reasonable estimate of the actuator energy density.

Derivation of Second Virial Coefficient Proportionality to Flory-Huggins Interaction Parameter
The second virial coefficient A_2 obtained from SLS and the Flory–Huggins interaction parameter χ are linked because both arise as coefficients of the quadratic term in the osmotic pressure expansion.[10]

The Debye-Zimm Equation (**Eq. 2**) is related to osmotic pressure (Π) since SLS measures the isothermal partial derivative with respect to concentration:

$$\frac{Kc}{R_\theta} = \frac{1}{RT}\left(\frac{\partial \Pi}{\partial c}\right)_T \quad \textbf{(Eq. S4)}$$

where R is the ideal gas constant and T is the temperature of the system.

Integration of **Eq. S4** with respect to c yields

$$\frac{\Pi}{RT} = \frac{c}{M_w} + A_2 c^2 + \cdots \quad \textbf{(Eq. S5)}$$

For a binary polymer solution, each lattice site is occupied by either polymer (ϕ) or solvent (1-ϕ). Thus, the Flory–Huggins free energy of mixing per lattice site is given by

$$\frac{\Delta G_{mix}}{RT} = n_w \ln(1-\phi) + n_p \ln(\phi) + \chi n_w \phi \quad \textbf{(Eq. S6)}$$

where n_w is the number of explicit water molecules and n_p is the number of polymer molecules.

The chemical potential of a component (μ_w) is defined as the partial derivative of the total Gibbs free energy with respect to the number of moles of that component:

$$\Lambda\mu_w = \left(\frac{\partial \Delta \mathrm{G}_{mix}}{\partial n_w}\right)_{T,P} \quad \textbf{(Eq. S7)}$$

The partial derivative of **Eq. S6** with respect to n_w gives

$$\frac{\Lambda\mu_w}{RT} = \ln(1-\phi) + \left(1-\frac{1}{N}\right)\phi + \chi\phi^2 \quad \textbf{(Eq. S8)}$$

where N is the degree of polymerization, such that the polymer occupies N lattice sites.

Osmotic pressure is related to the solvent chemical potential by

$$\Pi = -\frac{\Delta\mu_w}{V_m} \quad \textbf{(Eq. S9)}$$

where V_m is the molar volume of water. Finally, substituting **Eq. S6** into **Eq. S8** yields the expression:

$$\frac{\Pi}{RT} = -\frac{1}{V_m}\left[\ln(1-\phi) + \left(1-\frac{1}{N}\right)\phi + \chi\phi^2\right] \quad \textbf{(Eq. S10)}$$

Expanding the logarithmic term in **Eq. S10** yields the final Virial form:

$$\frac{\Pi}{RT} = \frac{1}{V_m}\left[\frac{\phi}{N} + \left(\frac{1}{2}-\chi\right)\phi^2 + \cdots\right] \quad \textbf{(Eq. S11)}$$

Using $\phi = c\bar{V}$ where $\bar{V}$ is the polymer specific volume, we can equate **Eq. S5** with **Eq. S11** and obtain the final relation between A_2 and χ:

$$A_2 = \frac{\bar{V}^2}{V_m}\left(\frac{1}{2}-\chi\right) \quad \textbf{(Eq. S12)}$$

Supplementary Figures

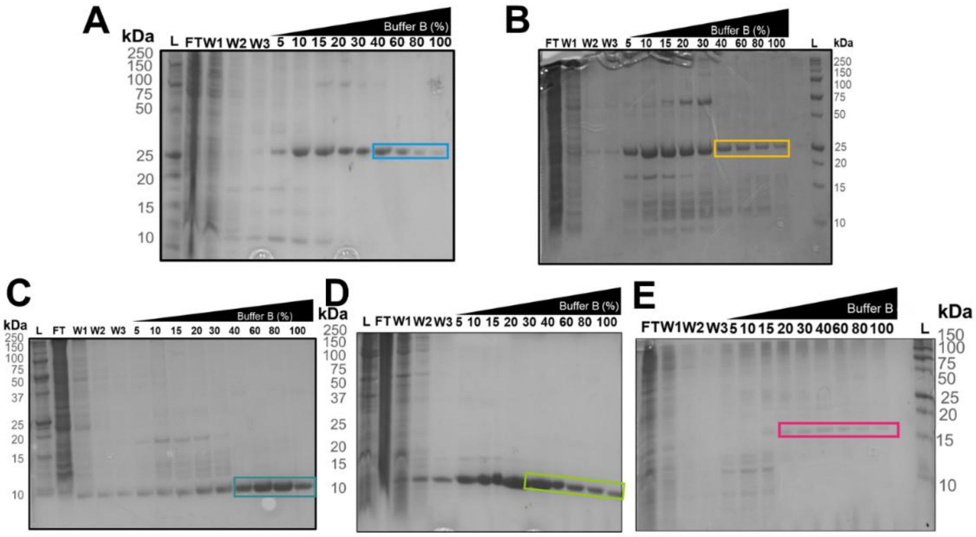

Figure S1. 12% SDS-PAGE confirmation of purified protein BCPs, specifically diblocks (**A**) CE and (**B**) $C_{L44A}E$, along with monoblocks (**C**) C, (**D**) C_{L44A}, and (**E**) E during immobilized metal affinity chromatography. Lane labeled L corresponds to a 10–250 kDa protein ladder with corresponding molecular weights denoted. Proteins were eluted from Ni-NTA agarose resin using an isocratic imidazole gradient generated by increasing proportions of Buffer B, ranging from 5 to 100% (i.e. 25 mM to 500 mM imidazole), with collected fractions highlighted with colored boxes.

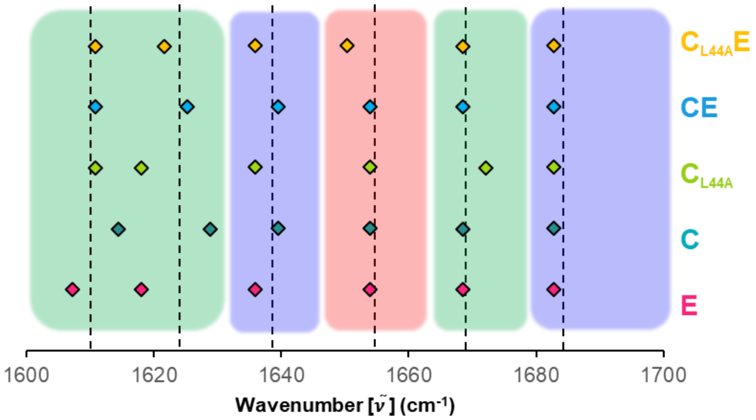

Figure S2. Second-derivative analysis of ATR-FTIR spectra highlights distinct subpeaks within the Amide I region ($\tilde{\nu}$=1,600–1,700 cm^{-1}). Characteristic ranges corresponding to α-helices ($\tilde{\nu}$= 1648–1660 cm^{-1}), β-sheets ($\tilde{\nu}$= 1625–1640 cm^{-1} and $\tilde{\nu}$= 1675–1695 cm^{-1}), and random coil structures ($\tilde{\nu}$= 1610–1628 cm^{-1}, 1640–1648 cm^{-1}, and 1660–1670 cm^{-1}) are outlined in red, blue, and green, respectively based on the empirical Jackson model.[11] Samples display major subpeaks (denoted by the black dotted lines) at $\tilde{\nu}$ = 1623, 1638, 1654, 1670, and 1683 cm^{-1} along with a minor feature near $\tilde{\nu}$ = 1610 cm^{-1} within the random-coil regime.

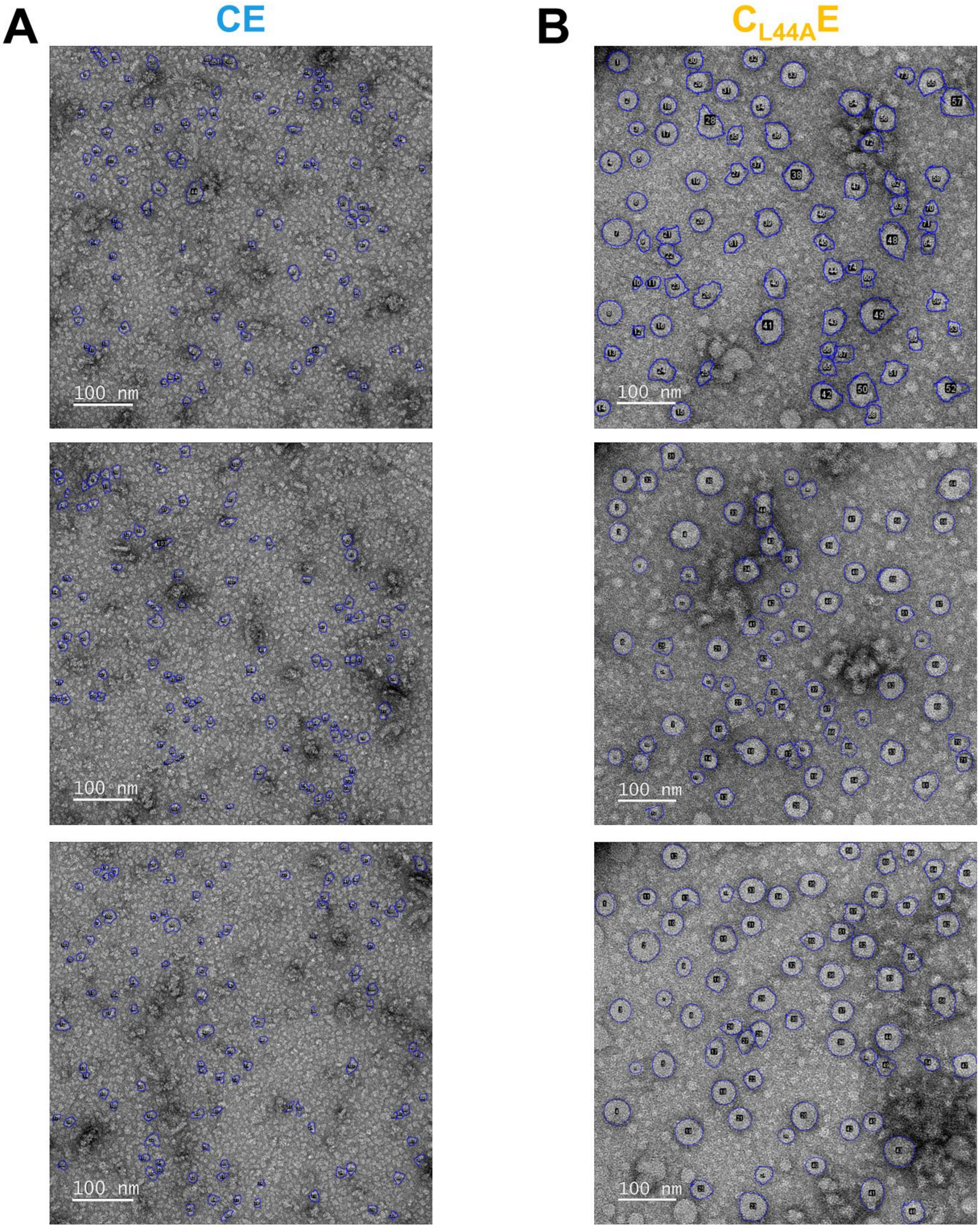

Figure S3. Additional TEM micrographs of (A) CE and (B) $C_{L44A}E$ micelles captured from three independent grid regions with ROIs drawn for morphological analysis. Multi-field sampling ensures reported dry particle Feret diameters are representative of bulk assembly populations.

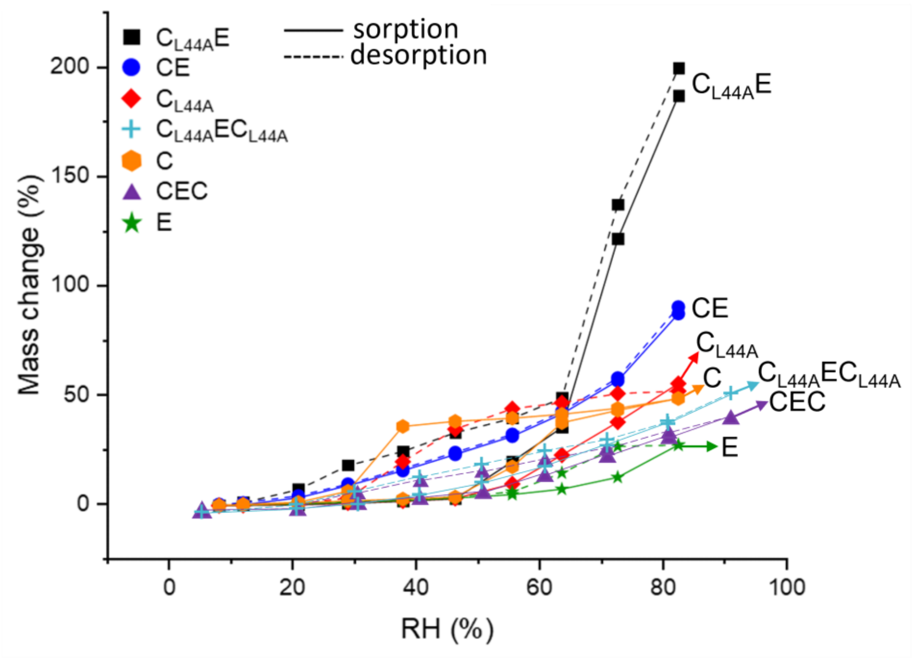

Figure S4. DVS equilibrium water sorption (solid lines) and desorption (dotted lines) isotherms for all protein BCPs to date, measured from 5–90% RH: $C_{L44A}E$ (black square), CE (blue circle), C_{L44A} (red diamond), $C_{L44A}EC_{L44A}$ (teal plus), C (orange hexagons), CEC (purple triangles), E (green stars). Data for CEC and $C_{L44A}EC_{L44A}$ were adapted and reproduced for comparison.[5]

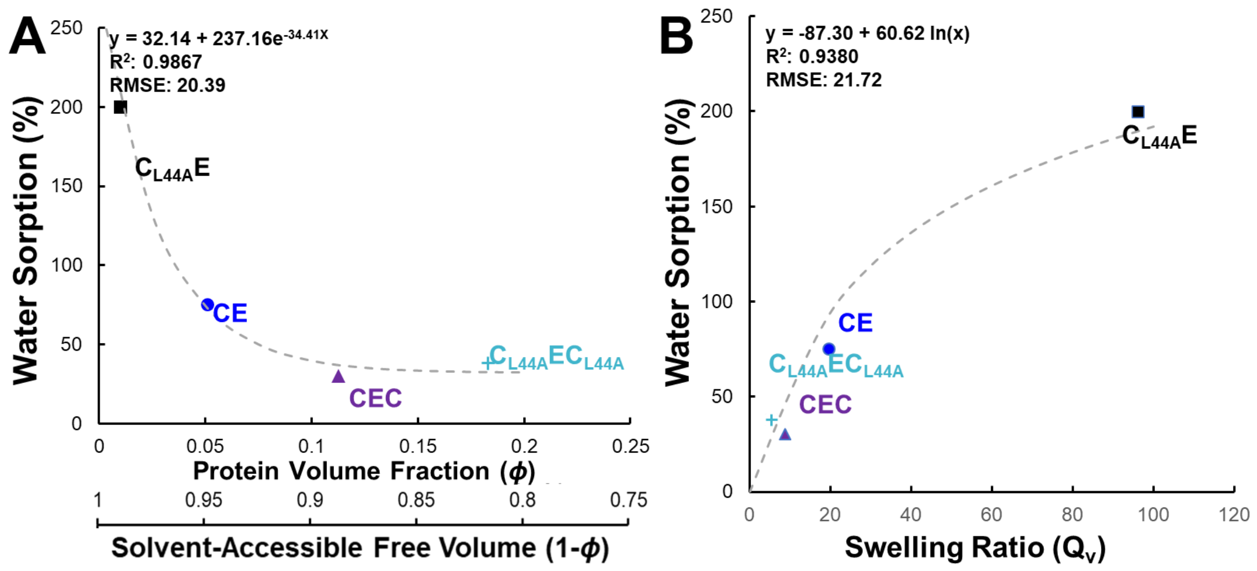

Figure S5. Correlative analysis between water sorption and the protein BCP series. (**A**) Water sorption as an exponential decay function of the protein BCP domain volume fraction or solvent-accessible free-volume, and **(B)** water sorption as a logarithmic function of the swelling ratio.

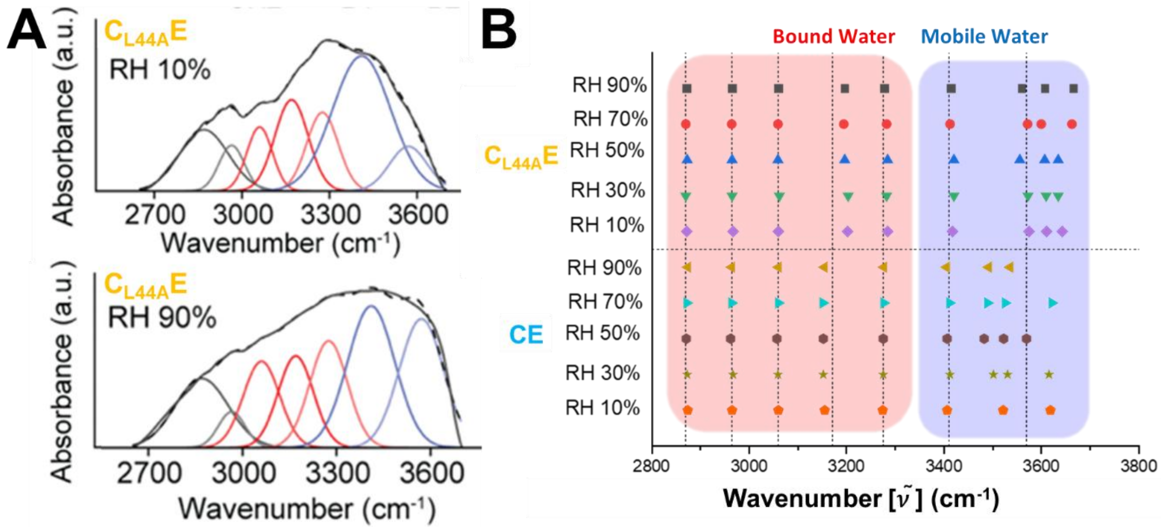

Figure S6. (**A**) Example spectral deconvolutions of the stretching region ($\tilde{\nu}$ = 2,700–3700 cm^{-1}) for $C_{L44A}E$ at 10% (top) and 90% RH (bottom). (**B**) Second-derivative analysis and bound/mobile water peak assignments following the Podbevšek model, where lower-wavenumber peaks ($\tilde{\nu}$ = 3000–3300 cm^{-1}) correspond to strongly H-bonded water associated with the protein BCP, whereas higher-wavenumber features ($\tilde{\nu}$ = 3410 and 3570 cm^{-1}) reflect weakly bound, bulk-like mobile water.[12] Adjacent peaks at 2870 and 2965 cm^{-1} arise from symmetric and asymmetric CH_3 stretching modes, respectively.[13]

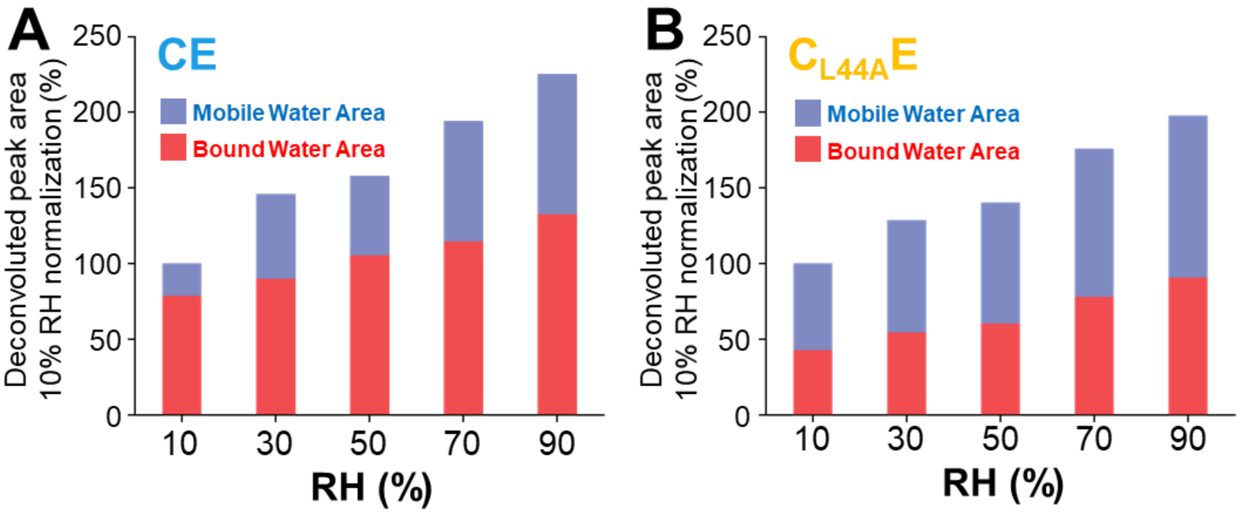

Figure S7. Relative growth of mobile and bound water deconvoluted from FTIR for (**A**) CE and (**B**) $C_{L44A}E$, normalized to the total area under the water peak at 10% RH for each BCP sample.

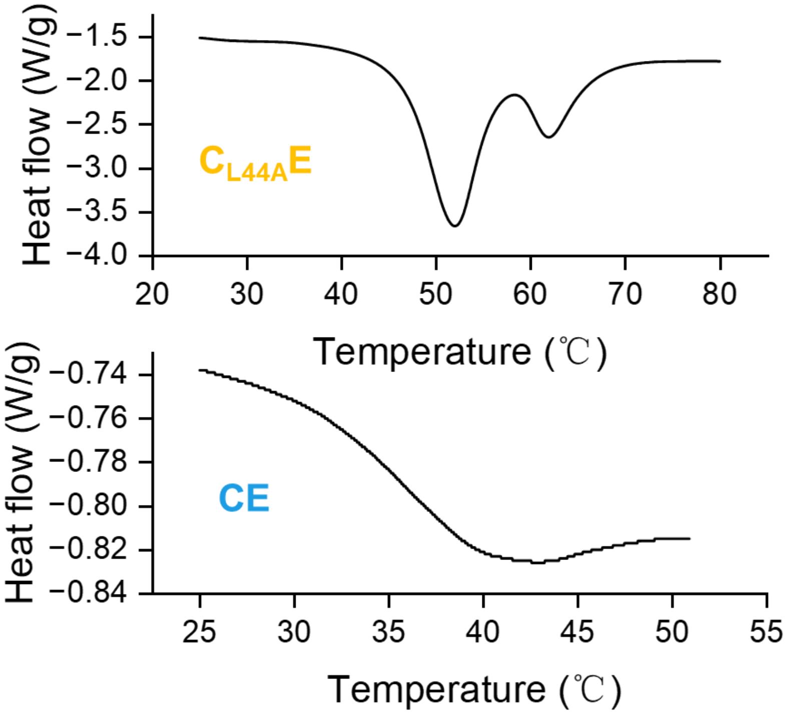

Figure S8. Magnified regions of the DSC thermograms for $C_{L44A}E$ and CE, emphasizing endothermic thermal transitions in the 20–80 °C range.

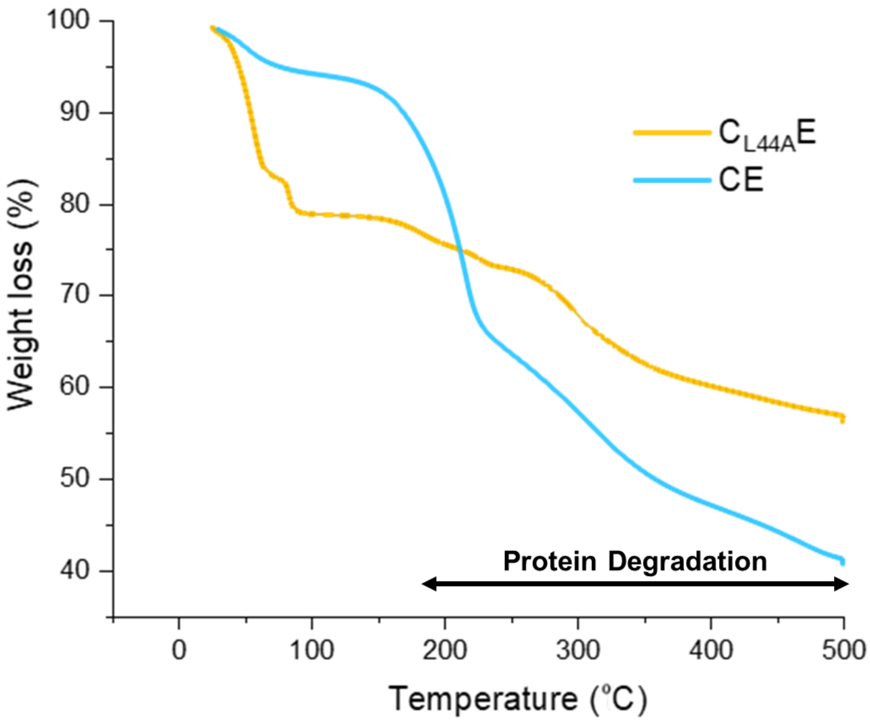

Figure S9. Extended TGA thermogram up to 500 ºC showing another mass loss event around ~250 ºC for CE and ~300 ºC for $C_{L44A}E$, consistent with thermal degradation.[14, 15]

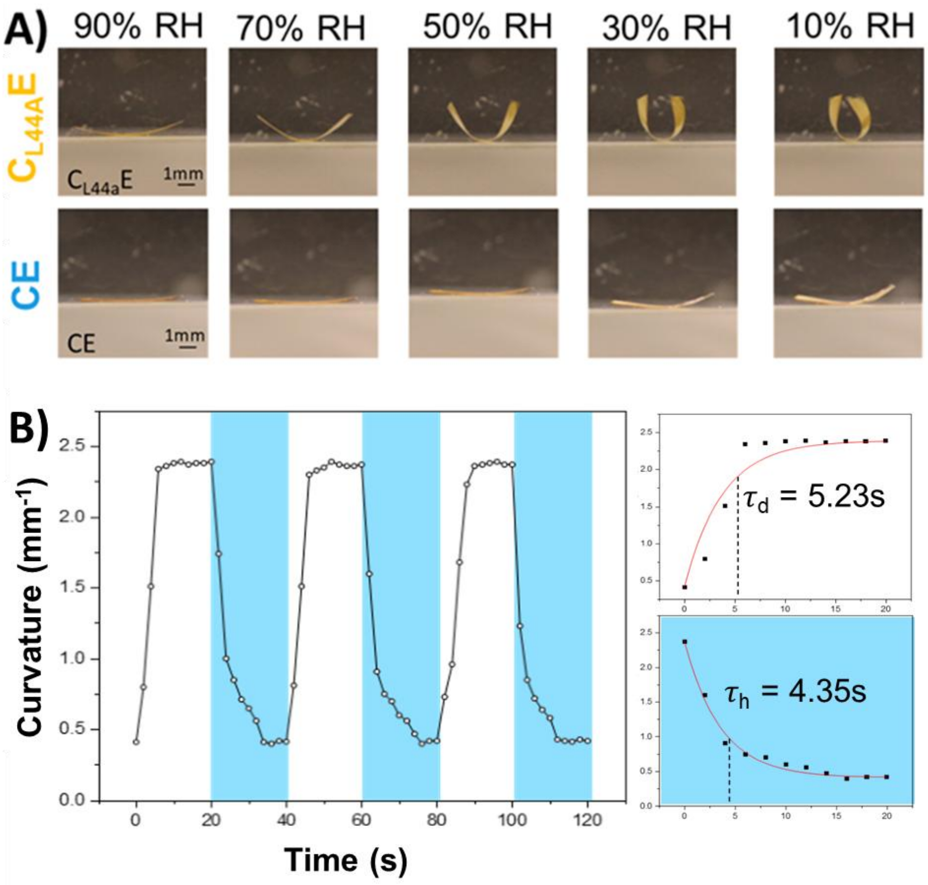

Figure S10. Reversible humidity cycling of diblock protein BCP/PI actuator films. (**A**) Representative still images showing the evolution of film curvature during RH cycling between 10% and 90% RH. (**B**) Curvature changes of $C_{L44A}E$/PI bilayers as a function of time that were used to extract actuation time constants for hydration (τ_H) and dehydration (τ_D). White regions denote dehydration from 10% to 90% RH, while blue regions indicate hydration from 90% to 10% RH. Three reversible cycles were performed over a total duration of 120 minutes.

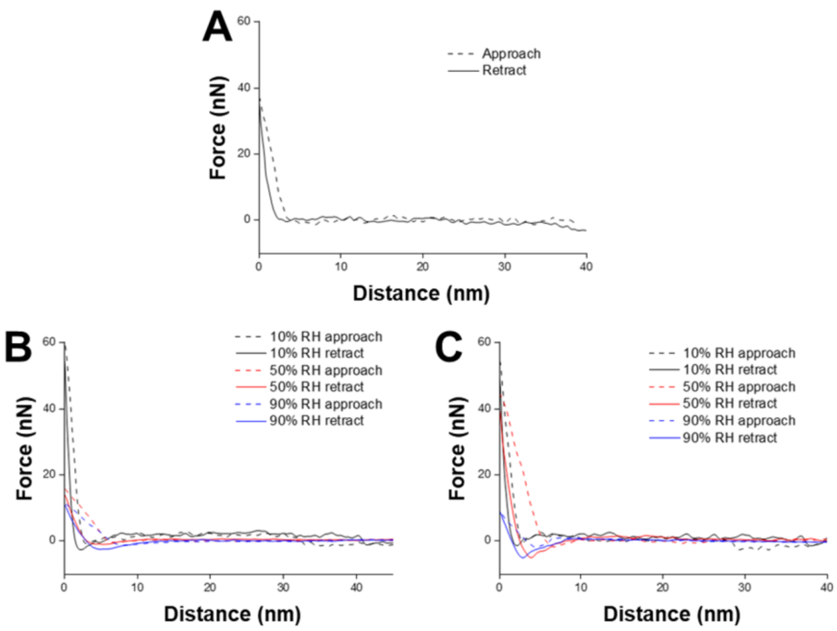

Figure. S11. Representative nanoindentation force-distance profiles for (**A**) polyimide (PI) solid support alone (**B**) CE films, and (**C**) C_{L44A}E films at 10%, 50%, and 90% RH. The measured Young's modulus for the bare PI films (2.6 ± 0.3GPa) agrees well with established literature values reported for polyimide.[16]

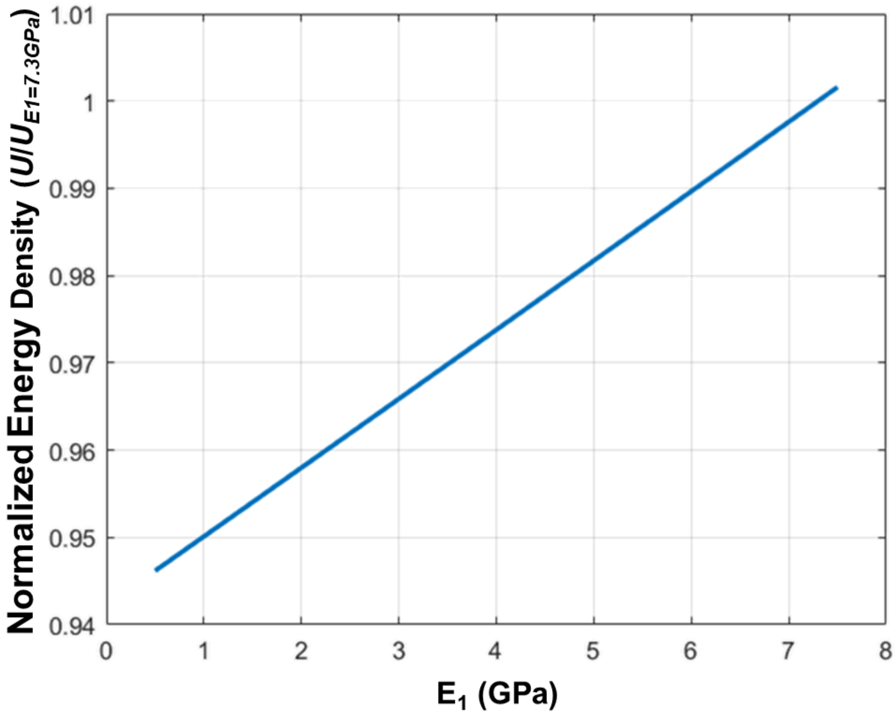

Figure S12. Sensitivity analysis of the WR actuator's energy density (U) to changes in the elastic modulus of the protein BCP layer (E_1).

Supplementary Tables

Table S1. Amino acid sequences for protein BCPs discussed in this work

Block Copolymer	Sequence
**CE**	MRGSH$_6$GSACELA(AT)$_6$AACG–**C**–LQA(AT)$_6$AVDKPIAASA–**E**–VPLEGSGTGAKLN
**C$_{L44A}$E**	MRGSH$_6$GSACELA(AT)$_6$AACG–C$_{L44A}$–LQA(AT)$_6$AVDKPIAASA–**E**–VPLEGSGTGAKLN
**E**	MRGSH$_6$GSKPIAASA–[**(VPGVG)$_2$VPGFG(VPGVG)$_2$**]$_5$–VPLEGSELGTPGRPAAKLN
**C**	MRGSH$_6$GSACELA(AT)$_6$AAC–GDLAPQMLRELQETNAA<u>L</u>QDVRELLRQQVKEITFLKNTVMESDASG–LQA(AT)$_6$AVDLQPSLIS
**C$_{L44A}$**	MRGSH$_6$GSACELA(AT)$_6$AAC–GDLAPQMLRELQETNAA<u>A</u>QDVRELLRQQVKEITFLKNTVMESDASG–LQA(AT)$_6$AVDLQPSLIS

Table S2. MALDI-ToF-MS Peak Parameters and Assignments

Ion Fragment	Absolute Intensity (a.i.)	Signal to Noise Ratio (S/N)	Full Width at Half Maximum (FWHM)	Theoretical Molecular Mass (Da)	Observed Molecular Mass (Da)	Obs–Theo (Da)
$[CE]^{+}$	410,720	811.1	42.066	22,323.61	22,328.29	4.68
$[CE]^{2+}$	162,302	31.5	36.930	11,161.81	11,143.42	-18.39
$[E]^{y+}$	96,559	96.9	10.747	10,263.05	10,259.23	-3.82
$[C]^{b+}$	14,877	12.1	53.406	12,060.56	12,055.51	-5.05
$[C_{L44A}E]^{+}$	514,951	168.8	405.951	22,281.53	22,276.84	-4.69
$[C_{L44A}E]^{2+}$	250,761	75.7	15.730	11,140.77	11,136.52	-4.25
$[C_{L44A}E]^{4+}$	37,019	5.3	74.988	5,570.39	5,551.83	-18.56
$[E]^{y+}$	68,534	55.9	11.304	10,263.05	10,258.62	-4.43
$[C_{L44A}]^{b+}$	31,408	6.0	148.268	12,018.48	12,016.83	-1.65
$[E]^{+}$	224,449	38.7	42.463	14,420.78	14,419.10	-1.68
$[C]^{+}$	425,224	8.1	94.928	10,721.98	10,718.09	-3.89
$2[C]^{+}$	25,671	8.8	187.111	21,443.96	21,446.22	2.26
$[C_{L44A}]^{+}$	601,027	6.3	111.155	10,679.90	10,679.71	-0.19
$2[C_{L44A}]^{+}$	21,602	5.7	253.814	21,359.80	21,352.06	-7.74

Table S3. Summary of $[\theta]_{MRE}$ values at CD spectral minima. Some constructs feature double minima (CE and C) and other only feature one distinct minima ($C_{L44A}E$, C_{L44A}, and E)

**Construct**	$\lambda_{min,1}$ (nm)	$[\theta]_{min,1}$ ($deg \cdot cm^2 \cdot dmol^{-1}$)	$\lambda_{min,2}$ (nm)	$[\theta]_{min,2}$ ($deg \cdot cm^2 \cdot dmol^{-1}$)
**CE**	208	-8,444.1 ± 468.9	222	-8,570.5 ± 459.9
**C**	208	-16,386.9 ± 4,494.5	222	-18,585.0 ± 5,915.4
**$C_{L44A}E$**	205	-5,242.5 ± 70.0	—	—
**C_{L44A}**	205	-9,471.1 ± 282.7	—	—
**E**	214	-8,039.9 ± 4,816.4	—	—

Supplementary References

1. Gunasekar, S. K.; Asnani, M.; Limbad, C.; Haghpanah, J. S.; Hom, W.; Barra, H.; Nanda, S.; Lu, M.; Montclare, J. K. N-Terminal Aliphatic Residues Dictate the Structure, Stability, Assembly, and Small Molecule Binding of the Coiled-Coil Region of Cartilage Oligomeric Matrix Protein. *Biochemistry* **2009,** *48* (36), 8559-8567. doi: 10.1021/bi900534r
2. Haghpanah, J. S.; Yuvienco, C.; Civay, D. E.; Barra, H.; Baker, P. J.; Khapli, S.; Voloshchuk, N.; Gunasekar, S. K.; Muthukumar, M.; Montclare, J. K. Artificial Protein Block Copolymers Blocks Comprising Two Distinct Self-Assembling Domains. *ChemBioChem* **2009,** *10* (17), 2733-2735. doi: 10.1002/cbic.200900539
3. Olsen, A. J.; Katyal, P.; Haghpanah, J. S.; Kubilius, M. B.; Li, R.; Schnabel, N. L.; O'Neill, S. C.; Wang, Y.; Dai, M.; Singh, N.; Tu, R. S.; Montclare, J. K. Protein Engineered Triblock Polymers Composed of Two SADs: Enhanced Mechanical Properties and Binding Abilities. *Biomacromolecules* **2018,** *19* (5), 1552-1561. doi: 10.1021/acs.biomac.7b01259
4. Dai, M.; Haghpanah, J.; Singh, N.; Roth, E. W.; Liang, A.; Tu, R. S.; Montclare, J. K. Artificial Protein Block Polymer Libraries Bearing Two SADs: Effects of Elastin Domain Repeats. *Biomacromolecules* **2011,** *12* (12), 4240-4246. doi: 10.1021/bm201083d
5. Kronenberg, J.; Jung, Y.; Chen, J.; Kulapurathazhe, M. J.; Britton, D.; Kim, S.; Chen, X.; Tu, R. S.; Montclare, J. K. Structure-Dependent Water Responsiveness of Protein Block Copolymers. *ACS Appl. Bio Mater.* **2024,** *7* (6), 3714-3720. doi: 10.1021/acsabm.4c00045
6. Studier, F. W. Protein production by auto-induction in high-density shaking cultures. *Protein Expr. Purif.* **2005,** *41* (1), 207-234. doi: 10.1016/j.pep.2005.01.016
7. Park, J.; Fagerquist, C. K. Exploring the fragmentation efficiency of proteins analyzed by MALDI-TOF-TOF tandem mass spectrometry using computational and statistical analyses. PLoS One **2024**, *19* (5), e0299287. doi: 10.1371/journal.pone.0299287
8. Wattenberg, A.; Organ, A. J.; Schneider, K.; Tyldesley, R.; Bordoli, R.; Bateman, R. H. Sequence dependent fragmentation of peptides generated by MALDI quadrupole time-of-flight (MALDI Q-TOF) mass spectrometry and its implications for protein identification *J. Am. Soc. Mass Spectrom.* **2002,** *13* (7), 772-783. doi: 10.1016/S1044-0305(02)00414-2
9. Stoney, G. G. The Tension of Metallic Films Deposited by Electrolysis. *Proc. R. Soc. Lond. A* **1909,** *82* (553), 172-175. url: www.jstor.org/stable/92886
10. Hiemenz, P. C.; Lodge, T. P. *Polymer Chemistry*, 2nd Ed.; Taylor & Francis: Oxfordshire, United Kingdom, 2007.
11. Jackson, M.; Mantsch, H. H. The Use and Misuse of FTIR Spectroscopy in the Determination of Protein Structure. *Crit. Rev. Biochem. Mol. Biol.* **1995,** *30* (2), 95-120. doi: 10.3109/10409239509085140
12. Podbevšek, D.; Jung, Y.; Khan, M. K.; Yu, H.; Tu, R. S.; Chen, X. The role of water mobility on water-responsive actuation of silk. *Nat. Commun.* **2024,** *15* (1), 8287. doi: 10.1038/s41467-024-52715-6
13. Šebek, J.; Knaanie, R.; Albee, B.; Potma, E. O.; Gerber, R. B. Spectroscopy of the C–H Stretching Vibrational Band in Selected Organic Molecules. *J. Phys. Chem. A* **2013,** *117* (32), 7442-7452. doi: 10.1021/jp4014674
14. Weiss, I. M.; Muth, C.; Drumm, R.; Kirchner, H. O. K. Thermal decomposition of the amino acids glycine, cysteine, aspartic acid, asparagine, glutamic acid, glutamine, arginine and histidine. *BMC Biophys.* **2018,** *11* (1), 2. doi: 10.1186/s13628-018-0042-4
15. Jagadeesh, D.; Jeevan Prasad Reddy, D.; Varada Rajulu, A. Preparation and Properties of Biodegradable Films from Wheat Protein Isolate. *J. Polym. Environ.* **2011,** *19* (1), 248-253. doi: 10.1007/s10924-010-0271-3
16. Poilane, C.; Delobelle, P.; Bornier, L.; Mounaix, P.; Melique, X.; Lippens, D. Determination of the mechanical properties of thin polyimide films deposited on a GaAs substrate by bulging and nanoindentation tests. *Mater. Sci. Eng. A* **1999**, *262* (1), 101-106. doi: 10.1016/S0921-5093(98)01002-8
